# Novel therapeutic agents in clinical trials: emerging approaches in cancer therapy

**DOI:** 10.1007/s12672-024-01195-7

**Published:** 2024-08-11

**Authors:** Deepak Chandra Joshi, Anurag Sharma, Sonima Prasad, Karishma Singh, Mayank Kumar, Kajal Sherawat, Hardeep Singh Tuli, Madhu Gupta

**Affiliations:** 1 Department of Pharmacy, School of Chemical Sciences and Pharmacy, Central University of Rajasthan, Bandar Sindri, Dist., Ajmer, Rajasthan India; 2 Invertis Institute of Pharmacy, Invertis University Bareilly Uttar Pradesh, Bareilly, India; 3 Chandigarh University, Ludhiana-Chandigarh State Highway, Gharuan, Mohali, Punjab 140413 India; 4 Institute of Pharmaceutical Sciences, Faculty of Engineering and Technology, University of Lucknow, Lucknow, India; 5 Himalayan Institute of Pharmacy, Road, Near Suketi Fossil Park, Kala Amb, Hamidpur, Himachal Pradesh India; 6 Meerut Institute of Technology, Meerut, Uttar Pradesh India; 7 Department of Bio-Sciences & Technology, Maharishi Markandeshwar Engineering College, Maharishi Markandeshwar (Deemed to Be University), Mullana, Ambala, India; 8 Department of Pharmaceutics, School of Pharmaceutical Sciences, Delhi Pharmaceutical Sciences and Research University, New Delhi, India

**Keywords:** Cancer, Cancer therapy, Checkpoint inhibitor, Kinase inhibitor, CAR-T cell therapy, Cancer vaccines, Epigenetic therapies

## Abstract

Novel therapeutic agents in clinical trials offer a paradigm shift in the approach to battling this prevalent and destructive disease, and the area of cancer therapy is on the precipice of a trans formative revolution. Despite the importance of tried-and-true cancer treatments like surgery, radiation, and chemotherapy, the disease continues to evolve and adapt, making new, more potent methods necessary. The field of cancer therapy is currently witnessing the emergence of a wide range of innovative approaches. Immunotherapy, including checkpoint inhibitors, CAR-T cell treatment, and cancer vaccines, utilizes the host’s immune system to selectively target and eradicate malignant cells while minimizing harm to normal tissue. The development of targeted medicines like kinase inhibitors and monoclonal antibodies has allowed for more targeted and less harmful approaches to treating cancer. With the help of genomics and molecular profiling, “precision medicine” customizes therapies to each patient’s unique genetic makeup to maximize therapeutic efficacy while minimizing unwanted side effects. Epigenetic therapies, metabolic interventions, radio-pharmaceuticals, and an increasing emphasis on combination therapy with synergistic effects further broaden the therapeutic landscape. Multiple-stage clinical trials are essential for determining the safety and efficacy of these novel drugs, allowing patients to gain access to novel treatments while also furthering scientific understanding. The future of cancer therapy is rife with promise, as the integration of artificial intelligence and big data has the potential to revolutionize early detection and prevention. Collaboration among researchers, and healthcare providers, and the active involvement of patients remain the bedrock of the ongoing battle against cancer. In conclusion, the dynamic and evolving landscape of cancer therapy provides hope for improved treatment outcomes, emphasizing a patient-centered, data-driven, and ethically grounded approach as we collectively strive towards a cancer-free world.

## Introduction

Cancer is a major widespread public health problem, causing a lot of deaths. Among the most prevalent kinds of cancer globally are breast cancer, lung cancer, liver cancer, gastric cancer, cervical cancer, and colorectal cancer [1]. There was a total of 609,640 deaths from all malignancies in the United States in recorded 2018, according to the American Cancer Society’s database on cancer occurrences, mortality, and survival [2]. In 2018, there were roughly 1,735,350 new instances of cancer diagnosed overall, excluding carcinoma (noninvasive cancer). Worrying statistics on cancer include an upward trend in cancer-related fatalities and diagnoses this year worldwide and projections that the global cancer burden will rise to almost 20 million people annually by 2025 [3, 4]. Consequently, cancer remains a worldwide health challenge that threatens people’s lives.

The current state of cancer therapy is marked by significant accomplishments as well as ongoing obstacles. For several decades, conventional treatment approaches, including surgery, radiation therapy, immunotherapy, hormone therapy, targeted therapy, and chemotherapy, have been widely employed as the primary strategies in combating this severe illness [5]. Although these therapies have been crucial in preserving several lives, they are not exempt from certain constraints [6]. They aren’t always effective against cancer, may have unpleasant side effects, and can’t be targeted specifically to cancer cells. Hence, it is imperative to comprehend the present condition of cancer treatment to fully grasp the pressing requirement for innovative approaches and fresh therapeutic agents [7].

The need for innovative therapeutic agents in the field of cancer therapy arises from the limitations of conventional methods. While many types of cancer exhibit positive responses to traditional therapy, several other forms of cancer persistently present substantial obstacles [8]. There is a growing trend among patients and healthcare professionals to explore alternative approaches that not only enhance the effectiveness of cancer treatment but also alleviate the physical and mental burden commonly associated with such therapy. The motivation to develop new therapeutic agents stems from the desire to discover treatment choices that are more efficient, specific, and less harmful, therefore offering cancer patients a sense of optimism and enhanced well-being [9].

## Traditional cancer therapies and their limitations

Cancer is a pervasive worldwide healthcare concern that accounts for approximately one in six of the total mortality globally. The prevalence of cancer in 2020 reached approximately 19.3 million, resulting in around 10 million deaths due to cancer. Cancer is an extremely complex disease that develops over time and worsens with a progressive loss of control over growth in many different areas [10]. Chemotherapy, radiation therapy, and surgical procedures are the three primary treatments that are being utilized in the treatment of cancer. The origins of chemotherapy may be traced back to the early twentieth century, however, its application in cancer treatment commenced in the 1930s. The German scientist Paul Ehrlich, with a specific focus on alkylating chemicals, was the one who first used the term “chemotherapy” to describe the process of treating diseases through the use of chemical approaches [11]. Surveys made during the two World Wars revealed that soldiers who were exposed to mustard gas exhibited reduced amounts of leukocytes. Consequently, Gilman employed nitrogen mustard as the inaugural chemotherapeutic drug for lymphoma treatment in 1943. In subsequent years, alkylating medicines, such as cyclophosphamide as well as chlorambucil, were chemically produced to combat cancer. Until the 1960s, surgery, and radiotherapy formed the fundamental approaches for treating solid tumors. As a result, the curability rates reached a plateau because the micrometastases were not being effectively managed [12, 13].

Conventional cancer treatments comprise a triad consisting of radiation, chemotherapy, and surgery. Among these, surgery is regarded as a valued foundation in the treatment of cancer. Physical removal of tumors or malignant tissues is its principal goal. In the case of localized malignancies, where the tumor is confined to a particular area, this technique demonstrates remarkable efficacy [14]. The efficacy of surgical interventions, nevertheless, is highly dependent on a multitude of variables, such as the tumor’s dimensions, its anatomical site, and the degree of metastasis. Notwithstanding its effectiveness, surgery is not devoid of obstacles. Although invasive procedures are crucial, they inherently bear the risk of complications, which may include infection or hemorrhaging. Moreover, prolonged recuperation periods are frequently required following surgical procedures, which negatively affects the patient's overall health [15]. One notable obstacle is that surgery may not be the most effective treatment option for malignancies that have metastasized extensively, thereby restricting its utility during the later phases of the illness. Surgery continues to be a critical component in the intricate realm of cancer treatment, providing targeted resolutions for a diverse array of malignancies. Its critical function is underscored by its efficacy, particularly when integrated with complementary therapies. Nevertheless, the difficulties linked to the invasive nature of the treatment and the constraints in effectively managing systemic malignancies underscore the persistent need for a comprehensive and varied strategy in cancer management [16]. Both researchers and clinicians are motivated to investigate novel approaches that can complement conventional treatments, thereby improving the overall effectiveness and reducing the difficulties faced by patients afflicted with this challenging illness.

Radiation therapy is a medical procedure that uses powerful radiation to specifically target and eradicate cancerous cells. This approach is very useful when cancer has spread to specific locations. Nonetheless, its non-specific targeting can cause collateral damage to surrounding healthy tissues, perhaps leading to long-term negative effects [17]. Furthermore, when dealing with large or deeply located tumors, the efficiency of radiation therapy may be impaired, posing difficulties for specific cancer types.

Chemotherapy is the injection of medications to slow the growth of cancer cells. While this technique has shown efficacy in treating numerous malignancies, its lack of specificity is a significant disadvantage [18]. Chemotherapy affects not just cancer cells but also healthy cells that divide fast, resulting in a variety of adverse effects. Furthermore, the development of medication resistance in cancer cells over time is a substantial challenge, necessitating changes in treatment regimens.

### Drawbacks and side effects of conventional treatments

Although conventional cancer treatments like surgery, chemotherapy, and radiation play a crucial role in fighting cancer, they do have drawbacks and associated adverse effects. These difficulties highlight the importance of continued study and the development of more sophisticated therapy techniques [19].One of the key drawbacks of these medicines is their non-targeted effects. Despite its accuracy in localized interventions, surgery has the potential to mistakenly injure surrounding healthy tissues, leading to problems. Similarly, the non-specific nature of radiation therapy can cause collateral damage to neighboring organs and tissues, contributing to long-term adverse effects including fibrosis or secondary malignancies in the treated region [20].

**Fig. 1:**
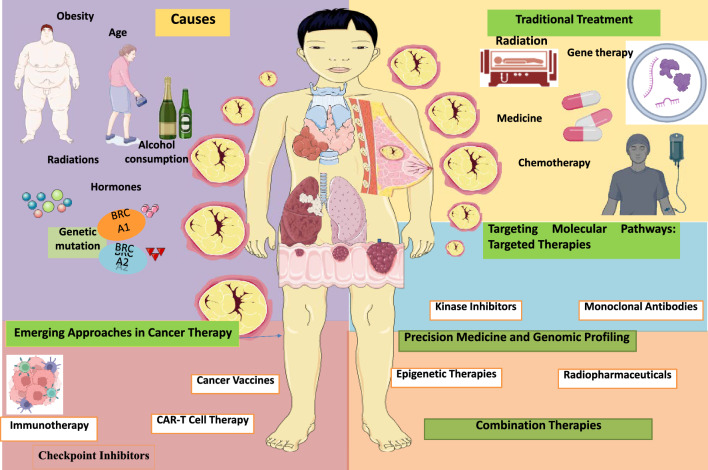
Different type of approaches in cancer therapy and causes

Chemotherapy, being a systemic treatment, has its own set of difficulties as shown in Fig. 1. Because of its non-selective action on rapidly proliferating cells, it affects both malignant and healthy cells, resulting in a variety of adverse effects [21]. Nausea, exhaustion, hair loss, and immunosuppression are significant side effects that frequently reduce the patient's quality of life during and after therapy. Furthermore, the evolution of drug resistance in cancer cells over time needs treatment plan revisions and may restrict chemotherapy's long-term efficacy. The effect on the patient's overall quality of life is a major source of worry. Traditional cancer therapies can be physically and emotionally draining, interfering with everyday activities and well-being. Long recovery times following surgery, enduring side effects during chemotherapy, and the possibility of long-term consequences from radiation therapy all contribute to the total load on patients [22].

## Emerging approaches in cancer therapy

### Harnessing the immune system: immunotherapy

Immunotherapy, which involves utilizing the immune system to fight cancer, is a revolutionary approach to cancer treatment that capitalizes on the body's natural defense systems. Checkpoint inhibitors, a key aspect of immunotherapy, have shown remarkable efficacy in several malignancies [23]. By inhibiting immunological checkpoints like cytotoxic T-lymphocyte-associated protein 4 (CTLA-4) and programmed cell death protein 1 (PD-1), these blockers activate the cytotoxic function of T-cells targeting cancer cells, resulting in long-lasting and effective responses in certain patients [24]. However, challenges such as immune-related adverse events, including autoimmune reactions and inflammatory responses, necessitate close monitoring and management during treatment. CAR-T cell treatment is a form of immunotherapy that includes modifying a patient’s T-cells to express chimeric antigen receptors (CARs), that can identify and destroy cancer cells [5]. This technique has shown exceptional efficacy, especially in the treatment of specific blood cancers, such as treatment-resistant B-cell acute lymphoblastic leukemia and non-Hodgkin lymphoma [25]. Despite the unprecedented therapeutic potential, CAR-T cell treatment holds immense therapeutic promise, it is also linked to potentially serious adverse effects, such as cytokines release syndrome and neurotoxicity, highlighting the need for improved safety measures and patient management protocols. Furthermore, the development of cancer vaccines has aimed to stimulate the immune system to recognize and eliminate tumor cells [26]. Various forms of cancer vaccines, such as those based on peptides, dendritic cells, and nucleic acids, are being investigated to stimulate strong and enduring immune responses against tumors [27]. While early successes have been observed, challenges related to optimizing vaccine efficacy, ensuring broader immune responses across diverse patient populations, and overcoming tumor immune evasion mechanisms remain significant hurdles in the successful implementation of cancer vaccines in clinical settings. Despite these challenges, ongoing research efforts continue to focus on refining immunotherapeutic approaches to achieve greater efficacy and broader applicability across various cancer types, offering new hope for patients with previously untreatable or difficult-to-treat malignancies. Moreover, the field of immunotherapy is rapidly evolving, with ongoing research aimed at identifying predictive biomarkers to better understand and anticipate patient responses to different immunotherapeutic approaches [28]. For instance, efforts are being made to elucidate the role of tumor mutational burden, programmed death-ligand 1 (PD-L1) expression, and other potential biomarkers in predicting patient responses to checkpoint inhibitors and other immunotherapies [29]. By identifying these predictive biomarkers, clinicians can better stratify patients and treatment approaches, therefore maximizing therapeutic results while minimizing potential negative consequences. Despite the remarkable progress in immunotherapy, challenges related to treatment resistance, tumor heterogeneity, and the development of immune evasion mechanisms by cancer cells continue to pose significant hurdles in the effective management of advanced or metastatic cancers [30]. To solve these problems, it is essential to adopt an extensive approach that integrates immunotherapy with other treatment methods, including targeted medicines and traditional treatments like radiation and chemotherapy. The synergistic potential of combination therapies holds promise in overcoming treatment resistance and enhancing the overall efficacy of cancer treatment regimens [31]. In the future, the research that is currently being conducted in the field of cancer immunotherapy intends to widen the use of these methods to a variety of cancers, such as solid tumors, while simultaneously improving the efficacy and safety of the immunotherapeutic therapies that are currently in use [32]. By unraveling the complex intricate relationship between the body's immune system and cancerous cells, researchers aim to develop more precise and effective immunotherapeutic strategies. These strategies have the potential to revolutionize cancer treatment, providing renewed optimism and better results for patients fighting different types of cancer. [33].

#### Checkpoint inhibitors

Checkpoint inhibitors have significantly transformed the field of cancer therapy by harnessing the immune system's potential to combat cancerous growths. They work by blocking the signaling pathways that regulate T-cell activation and response, effectively removing the brakes that prevent the immune system from recognizing and attacking cancer cells. One of the pivotal targets for checkpoint inhibitors is the cytotoxic T-lymphocyte-associated protein 4 (CTLA-4), a crucial immune checkpoint that down regulation T-cell activation [34, 35]. By inhibiting CTLA-4, these drugs promote sustained T-cell activation and proliferation, leading to enhanced anti-tumor immune responses. Similarly, programmed cell death protein 1 (PD-1) and its ligands, PD-L1 and PD-L2, are critical checkpoints that regulate T-cell responses within the tumor micro-environment [36]. Tumor cells often exploit the PD-1/PD-L1 pathway to evade immune surveillance, allowing them to proliferate unchecked. Checkpoint inhibitors that specifically target the PD-1/PD-L1 pathway interfere with the immune evasion strategy employed by cancer cells. This interference leads to the restoration of T-cell function, allowing the immune system to effectively identify and kill cancer cells. While checkpoint inhibitors have demonstrated unprecedented success in certain malignancies, including melanoma, non-small cell lung cancer, and renal cell carcinoma, their clinical benefits can vary widely among different cancer types and individual patients [37]. Furthermore, immunological-related side effects, resulting from the indiscriminate stimulation of the immune system’s response, can cause a range of autoimmune-like reactions that impact many organs, including the skin, endocrine, GIT, and liver glands [38]. Prompt recognition and management of these adverse events are crucial for ensuring patient safety and treatment continuation. Research efforts in the field of checkpoint inhibitors are also focused on identifying predictive biomarkers that can help determine patient responses to therapy. For instance, tumor mutational burden and PD-L1 expression levels have been explored as potential biomarkers for predicting the likelihood of a positive response to checkpoint inhibitor therapy. Integrating these biomarkers into clinical practice can aid in patient selection, enabling the delivery of more personalized and effective immunotherapy regimens [39]. Besides using a single treatment approach, current research is examining the possibility of integrating checkpoint inhibitors with various therapies, such as targeted treatments, chemotherapy, as well as radiation therapy, to improve the effectiveness of treatment and overcome resistance reactions. These combination approaches aim to leverage the complementary mechanisms of different therapies, ultimately leading to more robust and durable anti-tumor responses [40].

##### Checkpoint inhibitor of the CTLA-4 pathway supports the induction phase of anti-tumor T cell responses

**The CTLA-4 immune checkpoint**

The co-inhibitory receptor CTLA-4, which is increased early in the process of T cell activation, was the first negative modulator of T cell activation to be identified. Bound by the same ligands (CD80/CD86) that provide co-stimulatory signals through CD28 on T lymphocytes, CTLA-4 has the opposite effect. It mediates trans-endocytosis and degradation of the ligands needed for co-stimulation, and it delivers inhibitory signals that block T cell proliferation and secretion of IL-2. This leads to T cell tolerance through the induction of anergy [41, 42]. CTLA-4 adversely controls CD4 +and CD8 +T lymphocytes in various ways even though it is expressed on both types of cells. The priming of naïve cells is when CTLA-4 inhibits the responses of effector CD4 +T cells, but the memory CD8 +T cells, not the primary ones, are the ones that are most affected by CTLA-4 regulation. Moreover, CTLA-4 is essential to the immunosuppressive activity of CD4 +FOXP3 +regulatory T cells (Tregs) and is produced constitutively by them [43]. Thus, CTLA-4 activation on various T lymphocyte populations serves as an essential immune checkpoint that, in the end, reduces the acquisition of CD8 +T cell effector activity throughout primary and memory immunological responses. This is achieved through both direct and indirect processes.

**Immune checkpoint inhibitors targeting CTLA-4**

The co-inhibitory molecule CTLA-4 has gained interest as a potential target for treatments that attempt to increase the effector activity of T lymphocytes due to its function as a negative regulator of T-cell activation. Research in both animal models and human cancer patients has shown that blocking CTLA-4 with monoclonal antibodies improves the effector activity of CD4 +and CD8 +T cells, which is important for anti-tumor immunity [44]. In 2011, the U.S. Food and Drug Administration (FDA) authorized ipilimumab, the first immune checkpoint inhibitor, for use in cancer treatment, after a clinical trial showed that patients with unresectable stage III/IV melanoma had better survival rates after receiving treatment with a fully human IgG1κ anti-CTLA-4 monoclonal antibody [45]. After cutaneous melanoma patients have received total lymphadenectomy of tumor-involved regional lymph nodes and full resection, the FDA has authorized ipilimumab, marketed as Yervoy^®^ by Bristol-Myers Squibb, as an adjuvant treatment. Patients undergoing ipilimumab treatment had a 3-year survival rate of 22%, according to a pooled meta-analysis of 10 prospective and 2 retrospective trials evaluating the long-term survival of 1861 advanced melanoma patients; survival in some individuals extends close to 10 years. A contrast with the 3-year survival rates of metastatic melanoma patients treated with the FDA-approved chemotherapeutic drug dacarbazine highlights the importance of these results [46].

Despite its current monotherapy approval for melanoma, ipilimumab is undergoing research to explore its potential as a therapeutic intervention for a range of cancers, including nonsmall-cell lung carcinoma (NSCLC), prostate cancer, renal cell carcinoma (RCC), and others. While the clinical benefit against non-melanoma cancers has not been as great as in melanoma patients, there have been modest improvements in patient survival in certain subsets of non-melanoma cancer patients [47]. The results of ongoing trials are eagerly anticipated to determine the best way to use ipilimumab to treat different types of cancer. In addition, tremelimumab is a completely human IgG2 anti-CTLA-4 monoclonal antibody manufactured by AstraZeneca that is likewise being studied in clinical studies, even though ipilimumab is the only anti-CTLA-4 immune checkpoint inhibitor that has been approved by the FDA. Tremelimumab is now being studied in combination with durvalumab (an anti-PD-L1 immune checkpoint inhibitor covered below) or other regimens to determine whether it will have greater efficacy as part of combinatorial regimens, even though it has not improved patient survival as monotherapy in any trials to date [48].

##### Checkpoint blockade of the PD-1/PD-L1 *axis* maintains the effector phase of anti-tumor T-cell responses

**The PD-1 immune checkpoint**

Despite being identified as a molecule linked to programmed T cell death in 1992, PD-1 was not recognized as a co-inhibitory receptor that negatively controls T cell effector function until the autoimmune disease was seen in transgenic mice with PD-1 − / − T cells, which occurred several years later. Simultaneously, PDL1 and PD-L2 were identified as dual ligands for PD-1 and demonstrated to suppress T cell effector function after PD-1 activation. There is some evidence that PD-1 signaling can inhibit effector differentiation early in the induction phase of a T cell response and also promote the development and suppression of inducible Tregs [49, 50]. However, most data point to PD-1's role in suppressing effector that have already been differentiated in peripheral tissues, such as tumors, where this co-inhibitory receptor promotes T-cell exhaustion. T cells exhibit an increase and persistence of PD-1 expression after extended antigen exposure, as happens in chronic viral infection and developing malignancies, in contrast to the transitory expression that occurs after initial T cell activation. Over-expression of both PD-L1 (on different tumor cell types and tumor-associated APC) and PD-L2 (primarily on hematologic cancer cells and tumor-associated APC) is also caused by inflammatory stimuli and signaling networks that are frequently active in tumor micro-environments [36, 51].

Immunotherapies that seek to preserve or restore the effector function of anti-tumor T cells have found the PD-1 immune checkpoint pathway to be an appropriate target because these expression patterns lay the scene for suppression of PD-1 +tumor-infiltrating lymphocytes (TIL) [52].

**Immune checkpoint inhibitors targeting PD-1**

The development of PD-1-targeting ICB treatments for cancer patients was sparked by evidence from the aforementioned and similar research showing blocking the PD-1 pathway can improve tumor immune control and anti-tumor T cell reactivity. Nivolumab, a completely human IgG4κ monoclonal antibody (OPDIVO®, Bristol-Myers Squibb), was certified as the first PD-1-targeting ICB treatment for cancer by the FDA in 2014. This approval was derived from the results of the CheckMate-037 study, which showed that nivolumab had better objective response rates than the investigator's choice chemotherapy in patients with metastatic or unresectable melanoma whose cancers had advanced after being treated with ipilimumab, a ± BRAF inhibitor [53].

In a phase III trial comparing nivolumab to dacarbazine, the results showed that nivolumab improved objective response rate (40.0% versus 13.9%), progression-free survival (5.1 months versus 2.2 months), and overall survival (72.9% versus 42.1% at 1 year). As a result, nivolumab is now approved as first-line therapy for previously untreated melanoma that does not have a BRAF mutation. Nivolumab has now gained approval as an adjuvant treatment for patients with stage III/IV melanoma who are having their tumors surgically removed. In this particular situation, nivolumab demonstrated superior recurrence-free survival compared to ipilimumab [54, 55].

Compared to anti-CTLA-4 immune checkpoint inhibitors, nivolumab has demonstrated therapeutic benefit against a broader spectrum of malignancies, in addition to its effectiveness in treating melanoma. Nivolumab has been approved as either first- or second-line therapy for a variety of indications of squamous-cell carcinoma of the head and neck (SCCHN), advanced squamous-cell lung cancer (SCLC), and non-small cell lung cancer (NSCLC), and advanced renal cell carcinoma (RCC) [56]. Also, nivolumab has recently received expedited approval for the treatment of advanced urothelial cancer, advanced hepatocellular carcinoma (HCC), and metastatic colorectal cancer in patients with DNA mismatch repair-deficient or microsatellite instability-high, based on significant objective response rates in patients from Phase II trials [47–49]. Nivolumab was approved as the first checkpoint blockade inhibitor for the treatment of a hematological malignancy based on the findings of two separate Phase I/II trials that showed a combined objective response rate of 65% in patients with classical Hodgkin lymphoma who were treated with the drug [57].

Another anti-PD-1 immune checkpoint inhibitor that has drawn a lot of interest lately is pembrolizumab, a humanized IgG4κ monoclonal antibody sold by Merck under the brand name Keytruda^®^. Extended approval as frontline therapy for unresectable or metastatic melanoma in 2015 after two trials verified the survival benefits of this immune checkpoint inhibitor. Pembrolizumab was first approved in 2014 as an alternative to nivolumab for second-line treatment of patients whose disease had progressed after ipilimumab ± BRAF inhibitor therapy. With 2-year overall survival rates of 55% in pembrolizumab-treated patients versus 43% in ipilimumab-treated patients, a follow-up analysis of patients from the phase III KEYNOTE-006 study has shown lasting survival advantages of pembrolizumab therapy [54]. A similar story has played out for the approval of pembrolizumab for the treatment of metastatic non-small cell lung cancer. Accelerated approval was then extended after phase II/III trials revealed better overall and progression-free survival of patients with PD-L1 +tumors who received pembrolizumab compared to either docetaxel or platinum-based chemotherapy [58].

**Immune checkpoint inhibitors targeting PD-L1**

Targeting PDL1 with immune checkpoint inhibitors has shown to be a helpful strategy for enhancing the effector function of anti-tumor T cells in addition to disrupting the PD-1 immune checkpoint pathway by blocking PD-1 receptors on T lymphocytes. Roche Genentech’s fully humanized IgG1κ monoclonal antibody atezolizumab (Tecentriq^®^) was the first anti-PD-L1 monoclonal antibody to be approved for checkpoint blockade therapy in 2016. It was granted full approval for comparable indications of non-small cell lung cancer (NSCLC) and accelerated approval for the treatment of some indications of locally advanced or metastatic urothelial carcinoma. While second-line atezolizumab did not outperform chemotherapy in advanced urothelial carcinoma patients, atezolizumab did result in fewer and milder treatment-related adverse events than chemotherapy in these patients. The results of phase III trials evaluating the efficacy of atezolizumab as first-line therapy in cisplatin-ineligible urothelial carcinoma patients—for which accelerated approval was likewise granted in 2017—are currently awaited. Higher overall survival rates have been obtained with atezolizumab than with docetaxel in the setting of previously treated non-small cell lung cancer [59, 60].

Two more PD-L1 inhibitors, durvalumab (Imfinzi^®^, AstraZeneca/MedImmune) and avelumab (Bavencio^®^, EMD Sorono, Inc./Pfizer), have also been authorized in different contexts during the past year. Avelumab became the first FDA approved treatment for metastatic Merkel cell carcinoma and was approved for the treatment of metastatic urothelial carcinoma that has progressed after previous chemotherapy based on significant and durable objective response rates in phase I/II studies [64, 65]. Durvalumab's effect on progression-free survival in NSCLC patients (44.2% versus 27.0% 18-month progression-free survival rates in patients receiving durvalumab versus placebo) in the phase III PACIFIC trial [66] led to its full approval for treatment of stage III NSCLC that has not progressed following concurrent chemoradiotherapy. Durvalumab also received accelerated approval as second-line treatment for progressive metastatic urothelial carcinoma.

#### Chimeric antigen receptor (CAR) T cell therapy

Chimeric Antigen Receptor CAR-T cell therapy stands as a revolutionary advancement in cancer treatment, particularly for patients with limited options and poor prognoses. The process involves a sophisticated and personalized approach, starting with the extraction of the patient's T-cells, followed by the genetic modification of these cells using viral vectors to introduce CARs that can recognize and bind to specific tumor-associated antigens. Genetic engineering empowers T-cells to acquire exceptional specificity and potency in their ability to target cancerous cells, hence augmenting the immune system’s defense to fight malignant growth. CAR-T cell therapies have demonstrated remarkable efficacy in clinical settings, particularly in the management of specific forms of leukemia and lymphoma. It has achieved notable rates of response and, in certain instances, long-lasting remissions. Patients who have undergone multiple lines of traditional therapy and experienced relapses have reported significant improvements in their condition, often leading to prolonged periods of disease control and improved quality of life. However, the remarkable efficacy of CAR-T cell therapy is accompanied by the risk of potentially severe side effects, including cytokine release syndrome (CRS) and neurotoxicity. CRS, characterized by systemic inflammation and a surge of cytokine release, can lead to life-threatening complications, necessitating the implementation of strategies such as tocilizumab, an IL-6 receptor antagonist, to manage the symptoms and prevent further deterioration [61]. Neurotoxicity, although less common, remains a serious concern in CAR-T cell therapy. Its manifestations can range from mild confusion and aphasia to more severe symptoms such as seizures and cerebral edema. Early recognition and intervention are crucial in mitigating the risk of irreversible neurological damage and ensuring the best possible outcomes for patients undergoing this therapy. To address these challenges and broaden the therapeutic applications of CAR-T cell therapy, ongoing research is focused on refining the engineering of CAR-T cells, enhancing their persistence and efficacy, and minimizing the potential for off-target effects and treatment-related toxicities. Furthermore, investigations into novel targets beyond CD19, such as CD20, CD22, and BCMA, are underway to expand the reach of CAR-T cell therapy to other types of hematological malignancies and, potentially, solid tumors.

##### Chimeric antigen receptor (CAR) T cell therapy

Extracellular domain. Antigen recognition domain. The majority of CARs are single-chain variable fragments (ScFvs), which are genetically engineered by combining the light and heavy chain variable regions of a particular monoclonal antibody. These SCFs typically maintain the specificity and affinity of the original antibody. A linker, often a brief sequence of glycine-serine residues (averaging 15–20 amino acids in length), connects the two variable areas. By reducing the likelihood of linker interference with the activities of the linked domains, these residues provide flexibility. Additional forms of extracellular domains include “antibody TCRs,” which are characterized by the fusion of the Fab region of an antibody with the constant sections of the TCR [62]. This results in an increase in T cell activation due to the presence of entire TCR signaling components, which is a significant improvement over the traditional chimeric intracellular domain [63].

Furthermore, CARs that target autoantibody-producing B cells or allergen-specific B cells can be employed in the treatment of viral or autoimmune illnesses. These CARs have antigen recognition domains that are specific natural ligands, natural receptors, and B cell receptor ligands. Lastly, peptide/MHC (pMHC) complex-expressing CARs aimed at eliminating autoreactive T cells with pMHC-specific TCRs have the potential to eradicate undesirable clonal populations [64].

Spacer Domain is the transmembrane area linked to the extracellular domain by it, and the majority of it comes from IgG, CD8α, or CD28. It is essential to consider the length of the spacer since it has the potential to influence the activation of carrier cells. There is some evidence that CARs with shorter spacers activate T cells more effectively than those with longer ones [65]. There is a risk that it becomes more immunogenic, leading to in vivo depletion of carrier cells, or that it induces oligomerization of ScFv domains, promoting tonic CAR signaling; however, a longer spacer is necessary to guarantee antigenic binding by ScFv in cases where the target antigens are less accessible or closer to the membrane. The last step in making sure the CAR works well is to determine the ideal length based on how easily the target antigen can be accessed [66].

The transmembrane domain is primarily responsible for functioning as an anchor for the receptor to the cell membrane. It is responsible for connecting the extracellular domain to the intracellular domain. The hydrophobic alpha helix that makes up it crosses the membrane and is formed from CD4, CD8α, CD28, and CD3-ζ. Though this is the least researched area, certain findings suggest that for appropriate synapse formation or CAR surface expression, CARs require particular derivative areas within the transmembrane domain [65]. Intracellular Domain. The CAR T cells are activated by the signals that is provided downstream. The intracellular domain composition varies between the several generations of CARs that have been produced.

All that is included in first-generation CAR is the γ-chain of the TCR/CD3 complex or the γ-chain of the immunoglobulin E high-affinity receptor. Having three ITAMs (Immunoreceptor Tyrosine-based triggered Motif), the CAR α-chain can be triggered even in the absence of additional TCR-CD3 complex components [67]. Nevertheless, because T cells are exhausted early on, first-generation CAR has a limited anti-tumor effect. Knowing that a costimulatory signal is necessary for a full TCR activation, second and third-generation CARs were developed to imitate the second activation signal by including costimulatory molecule domains [68].

One costimulatory domain from molecules such as CD28, 4-1BB, CD27, DAP-12, OX40, or ICOS combined with the CD3-γ domain makes up a second-generation CAR. It is functionally important to choose and arrange these domains in the CAR construct since it could affect the carrier cells’ ability to proliferate and persist. As such, structures including appropriate intracellular domains can ensure and enhance the anti-tumor and survival properties of CAR cells [69]. Compared to the first generation, the second-generation CAR T cells exhibit better activation and proliferation as well as improved in vivo persistence. With two distinct costimulatory molecules next to the CD3-ζ domain, third-generation CAR has demonstrated high rates of proliferation, prolonged in vivo persistence, and decreased activation-induced cell death [70].

T lymphocyte activation requires a third signal (cytokine-dependent); thus a fourth-generation CAR was created that could induce cytokine signaling following antigen stimulation by combining the STAT-3 binding motif and the cytoplasmic domain of the IL-2 receptor β chain into the CD3-ζ and costimulatory cytoplasmic regions, producing CAR cells with increased persistence and anti-tumor effect [69].

##### The manufacturing of CAR T cells in clinical settings

To ensure the safety, sterility, purity, and potency of CAR T cells, quality control testing is conducted at every stage of the production process by Good Manufacturing Practices (GMP). CAR expression, in vitro evaluation of their functionality, lack of replication-competent viruses, purity of CAR T cell preparations (phenotypic markers), and lack of contaminants, such endotoxins or microorganisms (mycoplasma), must all be guaranteed before the product is released for infusion into patients [71].

Peripheral blood mononuclear cells (PBMC) are isolated from the patient, then T cells are enriched, activated, genetically transformed by viral or nonviral means, expanded, and then the patient is infused [72]. Although some organizations would rather enhance certain subpopulations like CD8, CD4, or central memory T cells, CD3 +cells are often employed extensively in clinical trials to produce CAR T cells. In particular, with retroviruses that exclusively transduce dividing cells, viral transmission requires T cell activation. Clinical trials employ two methods for activating T cells: anti-CD3/CD28 magnetic beads and anti-CD3 antibodies with IL-2. Following their construction, the CARs are transferred into the autologous T-cell genome using nonviral (transposons) or viral (lentiviral or retroviral) gene transfer mechanisms [73]. It takes extensive tropism and stability in cell lines to package CAR-expressing viral vectors. Viral vectors are generated by transfecting packaging cells with recombinant plasmids. Along with additional sequences needed for effective reverse transcription, RNA packaging, and integration, plasmids include the CAR construct. Despite their high reported efficiency of gene transfer, viral vectors are thought to be possibly carcinogenic and need for costly and extensive biosafety testing [73, 74].

The transposon/transposase system is a somewhat novel method for gene transfer; it has been utilized to electroporate CAR constructs into T cells. This system's benefit is its low cost of manufacture and simplicity, with a similar efficiency to the viral system. Its random insertion into the T cell genome raises the possibility of oncogenesis or disruption of other pertinent genes, though, much like viral gene delivery techniques. Finally, mRNAs brought into cells by endocytosis or electroporation are used by the messenger RNA transfer system. Although mRNA production in this method is temporary, genotoxicity and possible viral vector replication are minimized since no genomic integration takes place [75].

##### Clinical applications of CAR T cells

***CAR T cell for hematologic malignancies***

***Leukemias*** Leukemias are a class of diseases of the bone marrow and blood characterized by aberrant hematopoietic cells proliferating clonally. Patients with relapsed and refractory illness still have a dismal prognosis even with breakthroughs in treatment for acute and chronic leukemias, like chemotherapy/radiation conditioning regimens and allogeneic human hematopoietic stem cell transplantation (allo-HSCT) [76].

Making up over 80% of all pediatric acute leukemia, acute lymphoblastic leukemia (ALL) is the most prevalent malignancy in children. Though it is a deadly illness, ALL is the second most common acute leukemia in adults. Autologous anti-CD19 CAR T cell clinical studies have produced excellent remission rates in adult and pediatric r/r ALL patients of up to 93%. Crucially, minimum residual disease (MRD) negative remissions accounted for the bulk of them. Furthermore, remission has been seen in patients who had not previously undergone CAR T treatment or in patients who had relapsed following anti-CD19 CART therapy in an anti-CD22 CAR study [77].

Adult leukemia most commonly occurs as chronic lymphocytic leukemia (CLL). A poor prognosis awaits patients with relapsed and refractory CLL (r/r CLL). Promising outcomes from clinical trials including CAR T cells in r/r CLL patients include an overall response rate (ORR) of up to 75% and a complete response (CR) of up to 66%. Specifically, the effectiveness (CR) is lower for CLL than for ALL, hence novel CAR T or combination therapies are required to enhance these outcomes [78]. In this regard, new research has indicated better results with high ORR (83–100%), high CR (33–94%), and MRD negative (> 90%) in CLL patients treated with CAR T in conjunction with ibrutinib (Btk inhibitor) [79].

In conclusion, CAR T cell therapies have shown exceptional clinical efficacy in treating B-cell leukemias, with patients achieving unparalleled rates of full remission. Reducing treatment-related toxicities, such as cytokine release syndrome (CRS) and neurologic toxicity, is a major goal of these studies. The absence of particular target antigens has restricted the utility of CAR T cells in treating myeloid leukemias. Research in the lab has demonstrated that CAR T cells targeting either CD123 or CD33 can reduce cases of acute myeloid leukemia (AML). A CR was achieved by one patient and two patients demonstrated partial response after receiving anti-LeY CAR T cells in a phase I clinical study for r/r AML. The CAR T cells remained active for an extended period without causing any serious harm, according to this research [77, 78].

***Lymphomas*** There is a wide range of genetic, morphologic, clinical, and therapeutic variability among lymphomas. Both biological and clinical variables influence the prognosis. Although there are treatments available, the majority of people with r/r lymphoma will not recover [80]. The response rate to the next line of therapy was estimated to be 26% in a retrospective study of patients with refractory diffuse large B-cell lymphoma (DLBCL), with a complete response rate of 7%. In contrast, clinical trials utilizing CAR T cells for r/r DLBCL have achieved an overall response rate (ORR) of 50–85% and a CR of 40–50%. In addition, the ZUMA-1 study demonstrated an 83% overall response rate (ORR) and a 63% CR rate in patients with transformed follicular lymphoma (FL) or primary mediastinal B-cell lymphoma (PMBCL), a disease that is still incurable for most patients even with current therapies [78, 80].

***Novel CAR T cell therapies approaches to solid tumors***

Although CAR T cell therapy has shown promise in treating hematologic malignancies, its use in treating solid tumors is still in its early stages and has mixed outcomes. The tumor micro-environment (TME) seen in solid tumors not only inhibits T cell trafficking and infiltration but also reduces T cell function, proliferation, and persistence in the tumor. These represent the principal challenges faced in solid tumor CAR T cell therapy [80, 81].

Penetration into the Tumor Microenvironment (TME) is essential for the efficacy of CAR T cells. The metabolic environment inside the tumor is very hypoxic, inhibits T cell activation and effector function, and lowers T cell survival [82]. The physical barrier erected by stromal cells also prevents the entry of immune cells. Using genetically modified CAR T cells with enhanced capacity to break down the extracellular matrix or lower the number of tumor-infiltrating fibroblasts has been one approach to get beyond this issue. Furthermore, the aggressive expansion of cancer cells results in a nutritional depletion, including low levels of glucose and amino acids, which inhibit T cell activation and promote autophagy and alternative metabolic pathways that produce immunosuppressive mediators that cause T cell anergy and the development of regulatory T cells (Tregs) [83].

As chemokine receptors (CCR) expressed on cell surfaces and chemokines generated in the tumor microenvironment guide CAR T cell homing to the tumor, ensuring traffic to the tumor is another critical challenge in solid tumor treatment. Genetic reprogramming CAR T cells to express a CCR that increases tumor infiltration is one strategy that has been considered to enhance CAR homing, as other CCRs expressed by CAR T cells (e.g., CXCR3 or CCR5) do not presently complement the chemokine generated in the tumor [84].

The presence of various immunosuppressive leukocyte populations, such as tumor-associated macrophages (TAM), tumor-associated neutrophils (TAN), myeloid-derived suppressor cells (MDSC), and regulatory T cells (Tregs), which produce TGF-β, PGE2, indoleamine-2,3 dioxygenase (IDO), reactive oxygen/nitrogen species, tryptophan-2,3 dioxygenase (TDO), and arginase, hindering the recruitment and activation of cytotoxic T cells, is a significant limitation of CAR T treatment of solid tumors. Evidence also suggests that CAR T cells’ effector activity is impaired in this immunosuppressive environment [85]. Furthermore, invading lymphocytes often carry immune checkpoint molecules such as PD-1 and CTLA4, in addition to PD-L1 produced by cancer cells, which limits the anti-tumoral responses. Malignancies can indeed as be effectively treated by inhibiting the PD/PD-L1 axis, especially when combined with CAR T therapy. In the tumor micro-environment, particularly during the tumor’s escape phase, tumor cells can release several immunosuppressive cytokines, such as PGE2 and TGF-β [86]. To improve the efficiency of CAR T in vivo, it has been suggested to suppress the TGF-β signaling or activate PKA downstream of PGE2 [85].

Another factor that has limited the efficacy of CAR therapy in solid tumors is tumor-associated antigen (TAA) heterogeneity. Tumor cells expressing lesser amounts of the antigen or genetic mutation variations may escape CAR T therapy if the targeted eradication of cancer cells with higher immunogenicity occurs. In contrast, tumor cells expressing lower quantities of the antigen may survive [87].

#### Cancer vaccines

Cancer vaccinations are a hopeful approach in the field of cancer immunotherapy, aiming to activate the body’s immune system to selectively recognize and fight cancer cells. These vaccines can work through various mechanisms, including the introduction of tumor-specific antigens or the modulation of the immune response to better target and eliminate malignant cells [88, 89]. An approach to cancer vaccines involves utilizing antigens associated with tumors, which can be obtained from tumor cells or artificially produced in a laboratory. These antigens are administered to the patient, triggering an immune response that results in the activation of cytotoxic T-cells and the production of tumor-specific antibodies [90]. By targeting these antigens, the immune system is primed to recognize and destroy cancer cells, thereby inhibiting tumor growth and preventing metastasis. Dendritic cell-based vaccines represent another significant area of research in cancer vaccine development [91]. Dendritic cells have a vital function in delivering antigens to T-cells and triggering an immunological response. Within this framework, dendritic cells can be extracted from the patient's blood, subjected to tumor-specific antigens, and subsequently reintroduced into the patient [92]. This procedure stimulates the immune system, aiding in the identification and eradication of cancerous cells by cytotoxic T-cells. Moreover, mRNA-based vaccinations have garnered significant interest due to their capacity to elicit powerful immune responses targeting cancer cells [93]. These vaccines work by delivering synthetic mRNA encoding tumor-specific antigens into the patient’s cells, prompting the cells to produce these antigens and subsequently eliciting an immune response. The advantage of mRNA-based vaccines lies in their ability to induce both cytotoxic T-cell responses and antibody production, thereby enhancing the overall anti-tumor immune activity. While the development of cancer vaccines holds significant promise, several challenges persist in their clinical implementation [94]. These challenges include identifying the most effective antigens for a particular type of cancer, ensuring the delivery and presentation of these antigens to the immune system in a way that triggers a robust and targeted immune response, and overcoming the immunosuppressive micro-environment within the tumor that hinders the efficacy of the vaccine. To address these challenges, ongoing research efforts are focused on enhancing the immunogenicity of cancer vaccines, improving their specificity, and developing strategies to overcome tumor immune escape mechanisms. In addition, the investigation of combination strategies, such as the integration of cancer vaccines alongside various immunotherapies or typical therapies like radiation or chemo, aims to augment the overall defense against tumors and enhance therapeutic results for patients with diverse forms of cancer [95, 96].

#### Clinical trials with therapeutic vaccinations against human cancer viruses

*HBV and hepatitis C virus vaccines* Prevention vaccinations against hepatitis B virus (HBV) have been on the market for around 32 years, yet the virus continues to infect hundreds of millions of people throughout the world. Therapeutic vaccinations will so be required for some time to come [88]. There are now several therapeutic vaccinations available against the consequences of a chronic HBV infection [97]. Recombinant HBV proteins, DNA vaccines, recombinant virus vaccines, subviral particles, and immune complexes of IgG anti-HBsAg and HBV surface antigen (HBsAg) are among these vaccine platforms. Both DC ingestion of antigen and DC activation are facilitated by immune complex targeting via Fc receptors [98]. While a phase II experiment using these immune complexes made up of HBsAg and IgG Abs had encouraging results, a phase III randomized trial found no clinical or virological effect in individuals who were chronically infected with HBV. Therapeutic vaccines against hepatitis C virus (HCV) have utilized by and large the same vaccination platforms as those for vaccines against HBV [99]. So far, good immunogenicity data have been collected for some vaccines, but no efficacy data are available as of this writing. HBV and HCV do not contain oncogenic proteins that need to remain expressed in the transformed cells but rather cause hepatocellular cancer (HCC) by indirect mechanisms such as inflammatory events. This necessitates targeting persistent viral infection before malignant transformation, because HCC may not necessarily express viral proteins [99].

*EBV vaccines* While adoptive transfer of EBV-specific T cells is a classic success story for treating malignant illnesses caused by EBV, such as post-transplantation EBV-induced lymphomas, therapeutic immunization against EBV-related disorders is still comparatively new [100]. The EBV antigens EBNA-1 and LMP2 were included in a recombinant pox virus vector in a study that was published more than ten years ago and triggered CD4 +and CD8 +T cell responses against EBV [101]. In two separate phase I toxicity/immunogenicity studies conducted recently in patients with EBV-induced nasopharyngeal carcinoma (NPC) or other EBV-driven malignancies [65–67], the vaccine was well tolerated and generated antigen-specific T-cell responses. Though it does not stop the virus from spreading, an EBV vaccination may be able to stop the emergence of malignancies caused by the virus. It is remarkable how few groups actively pursue the development of efficient EBV vaccines given the large percentage of people who are continuously infected with the virus [102].

***Human T lymphotropic virus-1 vaccines*** One of the retroviruses known as human T lymphotropic virus-1 (HTLV-1) is responsible for the development of adult T cell leukemia/lymphoma (ATLL) or spastic paresis in a negligible percentage of persons who are consistently infected. In a recent trial, DCs loaded with HLA-A2 epitopes of the viral tax protein—which is implicated in both neoplastic transformation and production of spastic paresis—were used to immunize three ATLL patients who had previously received conventional chemotherapy [103].

***Merkel cell carcinoma virus vaccine*** Rare, quickly spreading skin cancer was only recently found to be caused by the Merkel cell carcinoma virus. Two oncogenic proteins, large T and small T, are produced by the virus. Adoptive transfer of T cells targeted against the virus in conjunction with intratumoral injection of IFNβ-1b or low-dose lesion irradiation might be an effective treatment for a patient with metastatic Merkel cell cancer [104]. Mice with small T–T-expressing B16 melanoma tumors in a preclinical therapeutic vaccination paradigm survived far longer following DNA immunization with a construct encoding small T than were animals treated with an empty vector. Considerable activation of T cell response was linked to small T vaccination. As small T is as much a foreign antigen to humans as it is to mice, this model is probably going to predict clinical effectiveness for this aggressive cancer type [105].

***HPV vaccines*** For the past ten years, 10- to 12-year-old girls have been eligible for preventive HPV vaccinations; yet, vaccination compliance is far from 100%, and the vaccines covering the high-risk HPV types (HPV16 and -18, which are linked to about 65% of all cervical cancers) have not yet been made available in the regions of the world where they are most needed [106]. Unfortunately, due to inadequate vaccination coverage and the huge cohort of infected persons from the pre-HPV vaccine era, many individuals in the Western world continue to be infected with high-risk HPV and face the risk of cervical cancer and HPV-positive head and neck cancer [106].

High-risk HPV, in particular HPV16, which accounts for around 50% of cervical carcinomas and 80% of HPV-positive head and neck cancers, has been the subject of most clinical trials utilizing therapeutic vaccinations in virally caused premalignant illness or cancer. Premalignant conditions include cervical intraepithelial neoplasia (CIN) and vulvar intraepithelial neoplasia (VIN) have so far shown the highest immunogenicity in terms of CD4 +and CD8 +T cell responses and clinical responses. Strong CD4 +T cell responses against several epitopes of the E6 and E7 oncogenic proteins and a somewhat less wide CD8 +T cell response were produced by vaccination with SLPs that overlap the whole sequence of these proteins given s.c. in Montanide ISA51 adjuvant. At 3 months following vaccination, over half of the patients had shown partial or whole regression of their lesions; at 12 months following the final immunization dose, this number increased even higher [107].

Furthermore, the clinical response and the potency of the T-cell immune response elicited by the vaccination correlated extremely significantly. This vaccine was interestingly far less immunogenic in patients with recurrent cervical cancer, and the induced T-cell responses stayed below the levels observed in VIN patients with clinical responses; in fact, vaccination did not provide a survival advantage over historical controls. This demonstrates one of the primary challenges with therapeutic cancer vaccination: T cell immunocompetence seems to be negatively impacted by cancer-associated changes in both systemic and local immunity. Application of Imiquimod ointment on the lesions followed by vaccination with TA-CIN, a fusion protein of HPV16 E6 and E7, and the viral capsid protein L2, produced outcomes in the treatment of VIN comparable to those with SLP immunization. Together with viral clearance, DNA vaccinations given by electroporation also produced a strong T-cell response and lesion regression. An alternative interesting vaccination is made from recombinant Listeria-E7 bacteria [108].

##### Peptide-based cancer vaccines

Cancer immunotherapy is now the fourth recommended cancer treatment after surgery, radiation, and chemotherapy. Generally speaking, the capacity of cancer immunotherapies to activate the host immune system against cancer cells divides them into two groups: “passive immunotherapies” and “active immunotherapies.” Cancer patients receiving immunologic drugs, including tumor-targeting monoclonal antibodies (mAbs) or adoptively transplanted T cells believed to have anti-tumor properties, are said to receive passive immunotherapies. By contrast, the introduction of antigens that elicit immune responses against cancer cells is what is meant to activate the host immune system in active cancer immunotherapies [109].

**Fig. 2:**
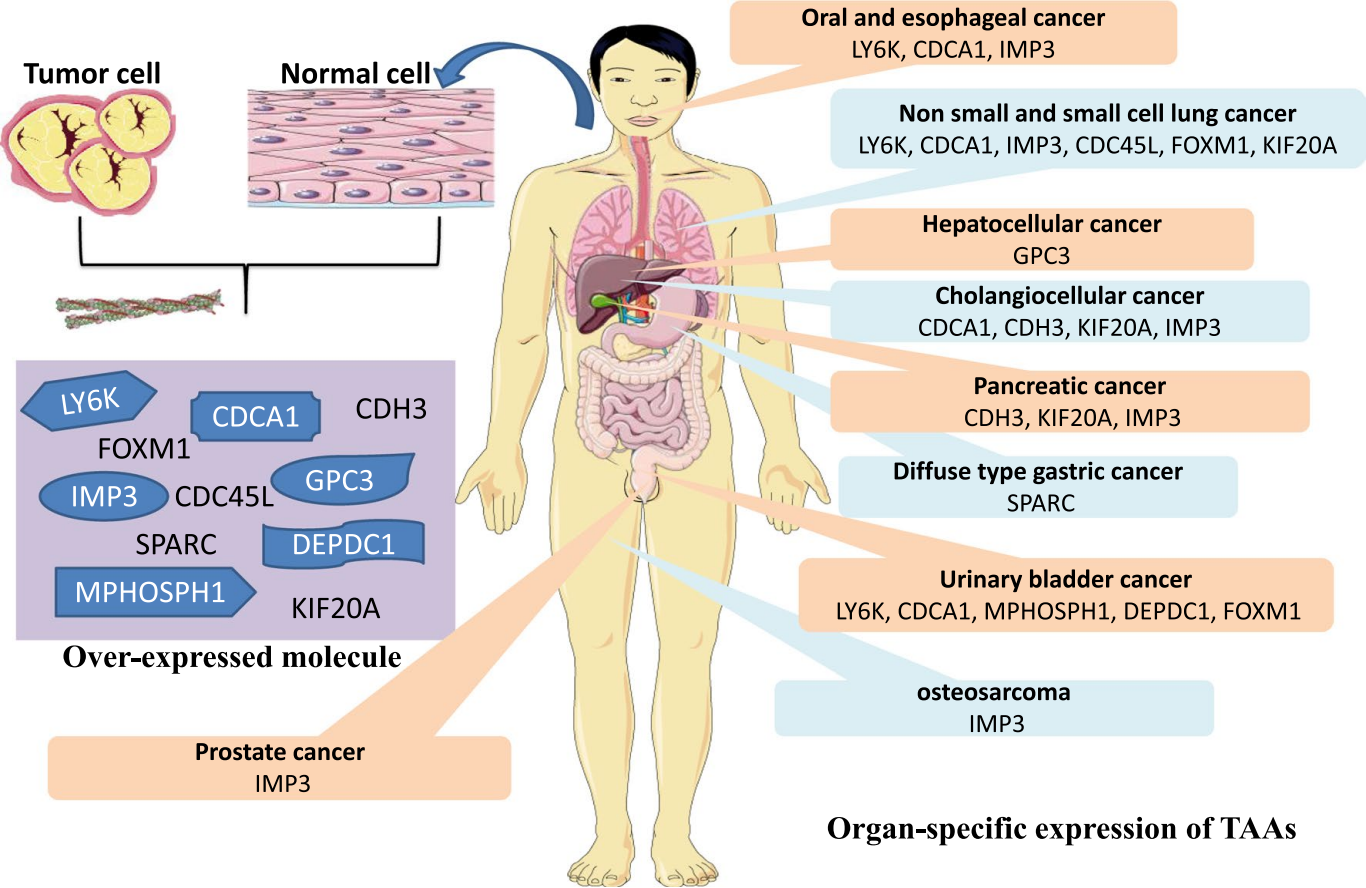
Targeted amino acid identification utilizing genome-wide cDNA microarray analysis for peptide-based cancer vaccines. Using a genome-wide cDNA microarray analysis, the gene expression patterns of both normal tissues and different tumor types were studied. Based on these findings, we can now pinpoint new tumor-associated antigens (TAAs) that are highly expressed in a wide range of cancerous tumors and exhibit features similar to CTAs and OFAs, making them excellent candidates for cancer immunotherapy

Vaccines for cancer are a form of “active cancer immunotherapy” that can stimulate the immune system to kill cancer cells specifically by exposing patients to synthetic peptides derived from tumor antigens (TAAs), recombinant TAA proteins, recombinant viral vectors encoding TAAs, dendritic cells (DCs) loaded with TAAs, or DNA/RNA-encoding TAAs [110]. Among the many cancer vaccine therapeutic modalities, peptide-based cancer vaccination stands out due to its low toxicity and ease of use, as it induces endogenous tumor-specific T-cell responses following administration of peptides derived from tumor-associated antigens (TAAs) as shown in Fig. 2.

**The recent identification of TAAs as potential cancer vaccine targets**

The development of safe and effective peptide-based cancer vaccines hinges on the identification of suitable TAAs to serve as immunotherapy targets for peptide therapies. The three properties listed below are necessary for the TAAs to be efficient molecular targets of this immunotherapy.

First, TAA expression patterns are particular to tumors. Tumor tissues must express TAAs specifically; normal tissues must not express TAAs or very little of them. Ideal TAAs in terms of preventing major autoimmune reactions include cancer-testis antigens (CTAs) and oncofetal antigens (OFAs), which are over-expressed in tumor tissues but not in normal tissues save for the testis and fetal organs.

And secondly, TAA immunogenicity (ICAA). The TAAs need to trigger T-cell-mediated immune responses in cancer patients and be able to be identified similarly to foreign antigens [111]. Third, features of TAAs that are cancerogenic. It is widely established that by downregulating particular TAAs or the production of human HLA class I molecules, cancer cells can directly avoid host T-cell identification. TAAs engaged in oncogenesis are thought to be hardly lost in the process of tumor growth, so they must possess physiologically carcinogenic properties to avoid a loss of targeted antigens during cancer vaccination [111].

Different TAAs have been found using genetic and immunological methods. Melanoma antigens A1 (MAGE-A1), gp100, or melanoma antigen recognized by T cells (MART-1/Melan-A) were found by screening cDNA expression libraries made from melanoma cell lines using tumor-reactive T-cell clones obtained from cancer patients as probes. Other TAAs, like New York esophageal squamous cell cancer 1 (NY-ESO-1) were identified by serological analysis of recombinant cDNA expression libraries using autologous patient antibodies as probes (SEREX method). Clinical trials including many of these TAAs as cancer immunotherapy targets are underway [111, 112].

In their study, Nakamura et al. used a genome-wide cDNA microarray analysis in conjunction with laser microbeam microdissection to isolate tumor tissues and compare them to normal tissues in order to determine gene expression patterns. With this approach, several cancer-specific, immunogenic, and oncogenic novel TAAs have been shown to be perfect targets for cancer immunotherapy [113]. Hepatocellular, esophageal, lung, pancreatic, and urinary bladder cancers; head and neck cancers (HNCs); and numerous other malignancies are among the many cancer tissues where these TAAs are often over-expressed and exhibit features of CTAs or OFAs [114].

### Targeting molecular pathways: targeted therapies

Targeted therapies in cancer treatment are designed to specifically interfere with the molecular pathways and processes that are critical for tumor growth and progression while minimizing damage to normal cells [115]. These therapies have significantly transformed the landscape of cancer treatment, offering more precise and effective alternatives to traditional chemotherapy and radiation therapy [116, 117]. One important category of targeted therapies involves kinase inhibitors, which work by blocking the activity of specific kinases that play a crucial role in the proliferation and survival of cancer cells. For instance, tyrosine kinase inhibitors (TKIs) target tyrosine kinases that are often overactive in certain types of cancer, including chronic myeloid leukemia (CML), non-small cell lung cancer (NSCLC), and gastrointestinal stromal tumors (GIST) [118, 119]. By inhibiting these kinases, targeted therapies can effectively disrupt the signaling pathways that promote cancer cell growth and division, leading to tumor regression and improved patient outcomes. Monoclonal antibodies represent another key class of targeted therapies, functioning by selectively binding to specific proteins expressed on the surface of cancer cells. By binding to these proteins, monoclonal antibodies can trigger immune-mediated cytotoxicity, block cell signaling pathways, or deliver cytotoxic agents directly to the cancer cells, ultimately leading to their destruction [120, 121]. Notable examples of monoclonal antibodies in cancer treatment include those targeting HER2 in breast cancer and EGFR in colorectal cancer, among others. Despite the remarkable success of targeted therapies, the development of treatment resistance remains a significant challenge [122, 123]. Cancer cells can acquire mutations or alternative signaling pathways that enable them to evade the effects of targeted therapies, leading to disease progression and relapse. To overcome this challenge, ongoing research efforts are focused on elucidating the mechanisms of resistance, identifying predictive biomarkers, and developing novel combination strategies that can enhance the efficacy of targeted therapies and delay the emergence of resistance. Furthermore, the integration of precision medicine and genomic profiling has played a crucial role in guiding the selection of appropriate targeted therapies for individual patients [124]. By identifying specific genetic alterations and molecular markers that drive tumor growth, clinicians can tailor treatment regimens to target these specific molecular aberrations, thereby maximizing the therapeutic benefits and minimizing the risk of adverse effects [125].

#### Kinase inhibitors

Kinase antagonists are an important type of targeted treatment used in cancer treatment. They are specifically designed to interrupt the abnormal signaling pathways that influence the growth and progression of tumors. Kinases are enzymatic proteins that have crucial functions in cellular communication and control, encompassing activities like cell growth, specialization, and programmed cell death [126]. In cancer, dysregulation of kinase activity often results in uncontrolled cell growth and survival, making kinases attractive targets for therapeutic intervention. Tyrosine kinase inhibitors (TKIs) constitute a significant subgroup of kinase inhibitors that have demonstrated remarkable efficacy in various cancer types [127]. These inhibitors function by binding to the ATP-binding site of tyrosine kinases, thereby preventing the transfer of phosphate groups to downstream signaling molecules and interfering with critical cellular processes necessary for tumor cell survival and proliferation. Imatinib, for instance, is a first-generation TKI that has exhibited exceptional success in the treatment of chronic myeloid leukemia (CML) and gastrointestinal stromal tumors (GIST). By specifically targeting the BCR-ABL fusion protein in CML and the KIT receptor tyrosine kinase in GIST, imatinib effectively inhibits the aberrant kinase activity, leading to the inhibition of cell proliferation and induction of apoptosis [128]. Furthermore, the development of second and third-generation TKIs has enabled the targeting of specific resistance mutations that emerge following treatment with first-generation inhibitors. These next-generation inhibitors exhibit improved binding affinity and selectivity for the target kinases, allowing for more potent and sustained inhibition of aberrant signaling pathways [129]. Examples include dasatinib and nilotinib, which have demonstrated efficacy in overcoming resistance mutations in patients with CML who have developed resistance to imatinib. Despite their successes, the use of kinase inhibitors can be associated with certain challenges, including the development of resistance mechanisms, off-target effects, and adverse events [130]. The emergence of secondary mutations or the activation of alternative signaling pathways can lead to the acquisition of resistance to kinase inhibitors, ultimately resulting in disease progression and treatment failure. To address these challenges, ongoing research efforts are focused on developing combination strategies that combine kinase inhibitors with other targeted therapies or conventional treatments to enhance their efficacy and delay the onset of resistance [131].

##### Kinase inhibitor binding sites

Protein kinases are characterized by their capacity to facilitate the transfer of the last phosphate group from ATP to substrates that typically contain a serine, threonine, or tyrosine residue. These components usually have a preserved arrangement of secondary structures, organized into 12 subdomains. These subdomains form a catalytic core structure with two lobes, and ATP binds in a narrow crevice between the lobes [132]. ATP attaches to the cleft via creating hydrogen bonds with the kinase ‘hinge’, which is the part that joins the amino- and carboxy-terminal kinase domains. The ribose and triphosphate groups of ATP form bonds within a hydrophilic channel that extends to the substrate binding site. This channel contains conserved residues that play a crucial role in catalysis. Every kinase possesses a preserved activation loop that plays a crucial role in controlling kinase activity. This loop is characterized by conserved DFG and APE motifs, which are represented by one-letter amino acid abbreviations at the beginning and end of the loop, respectively [133]. The activation loop can adopt a wide range of conformations, ranging from a catalytically competent and typically phosphorylated conformer to an “inactive” conformer where the activation loop obstructs the substrate binding site. The majority of kinase inhibitors that have been identified so far are ATP-competitive and make one to three hydrogen bonds with the amino acids in the hinge region of the target kinase. This allows them to imitate the hydrogen bonds that are typically generated by the adenine ring of ATP [134]. Most kinase inhibitors do not target the ribose binding site, except AZD0530, a unique inhibitor that targets both Src and Abl kinases. Additionally, these inhibitors do not target the triphosphate binding site of ATP [134, 135].

***Type 1 inhibitors-*** The majority of ATP-competitive inhibitors belong to this kind of inhibitor, which specifically binds to the active conformation of the kinase. This conformation is known to facilitate phosphotransfer33. The prevalence of type 1 inhibitors may be attributed to the fact that numerous compounds have been identified utilizing enzymatic assays that involved the introduction of kinases in their active conformation. Additionally, many kinase inhibitors have been produced to imitate ATP and each other. Type 1 inhibitors generally comprise a heterocyclic ring system that occupies the purine binding site. This ring system acts as a framework for side chains that occupy the nearby hydrophobic regions I and II.

***Type 2 inhibitors-*** In contrast, type 2 kinase inhibitors specifically identify and bind to the kinase's inactive conformation. The conformation that type 2 inhibitors recognize is commonly known as DFG-out due to the rearrangement of this motif. The repositioning of the activation loop into the DFG-out conformation reveals a supplementary hydrophobic binding site that is located right next to the ATP binding site. The initial finding that inhibitors like imatinib and sorafenib bind in the type 2 conformation was accidental, but further examination of various co-crystal structures of type 2 kinase inhibitors has shown that they all possess a similar pharmacophore and utilize a conserved group of hydrogen bonds [136]. Approved type 2 inhibitors by the US Food and Drug Administration include imatinib and nilotinib, which inhibit ABL1, KIT, and platelet-derived growth factor receptor (PDGFR), as well as sorafenib, which inhibits KIT, PDGFR, and Raf. The presence of inhibitors in type 2 kinase co-crystal structures reveals that the activation loop undergoes a conformational rearrangement that is stabilized by the inhibitors. This indicates that the active site of the kinase is highly flexible and can adapt to different inhibitors. Type II kinase inhibitors are not the only ones capable of causing significant changes in molecular structure. For instance, the type I inhibitor PIK-39 shows a preference for PI3Kγ compared to other PI3K isoforms by causing a rearrangement of the side chain of M804. This rearrangement creates a new pocket at the entrance of the kinase active site, similar to the conformation observed in DFG-out kinases [137]. While the methionine residue that PIK-39 binds with is present in other PI3Ks, the selectivity for PI3Kγ is achieved because only this specific isoform allows for a conformational rearrangement mediated by the inhibitor [138].

***Allosteric inhibitors-*** The third category of chemicals attaches to a location outside of the ATP-binding site, known as an allosteric site, and adjusts the activity of the kinase in an allosteric way. Inhibitors in this category have exceptional kinase selectivity as they target specific binding sites and regulatory mechanisms unique to each kinase. CI-1040 is the most extensively studied allosteric kinase inhibitor. It works by occupying a pocket next to the ATP binding site, hence inhibiting the activity of MeK1 and MeK2 [139]. Additional instances encompass GnF2, which attaches to the myristate binding site of BCR–ABL1; the Akt-I-1, an inhibitor of Akt that relies on the pleckstrin homology domain; and BMS-345541, an inhibitor of IKK (inhibitor of nuclear factor-κB kinase). Additional allosteric activators of kinase activity have been identified, such as RO0281675 and its analogues44 that stimulate glucokinase, as well as AICAR45 and A-769662, which activate AMP-activated protein kinase [140]. It is expected that more allosteric inhibitors will be discovered in the future due to the increased focus on cell-based assays that study kinases in their natural environment. This approach offers the benefit of enabling the identification of chemicals that may necessitate an auxiliary protein for proper functioning. For example- The necessity of the mTOR inhibitor rapamycin for the intracellular protein FKBP1A would not have been identified in a biochemical mTOR assay [141].

***Covalent inhibitors-*** The fourth category of kinase inhibitors consists of compounds that can establish an irreversible, covalent connection with the active site of the kinase. This interaction often occurs by interacting with a nucleophilic cysteine residue. The highly advanced irreversible kinase inhibitors, HKI-272 and CL-387785, were specifically designed to target a very uncommon cysteine residue positioned at the edge of the ATP binding region of the epidermal growth factor receptor (eGFR) [142]. The compounds were deliberately created by adding an electrophile, which reacts with the electron-rich sulfur found in the cysteine residue, to the well-known eGFR-selective 4-anilinoquinazoline and 4-anilinoquinoline-3-carbonitrile structures [143]. By utilizing the established structures of 4-anilinoquinazolines bound to eGFR, it was feasible to anticipate the most favorable location for electrophile bonding. The inhibitors produced undergo a Michael addition reaction, in which the cysteine residue (C773 in eGFR or C805 in eRBB2) that is exposed to the solvent forms a covalent bond with the inhibitor. This bond gives the inhibitor infinite affinity for the ATP binding site [144]. Consequently, the inhibitor permanently prevents the binding of ATP to the kinase, causing the kinase to become inactive. Currently, there are five eGFR kinase inhibitors undergoing evaluation in clinical studies for lung cancer.

Irreversible kinase inhibitors have been specifically developed to target vascular endothelial growth factor receptor 2 (VeGFR2), the Tec family kinase BTK, and RSK. Furthermore, there are naturally existing cytotoxic chemicals that have developed the ability to permanently alter kinase cysteine residues in this manner [145]. The compound hypothemycin, which is a type of resorcylic acid lactone polyketide, was found in the fungus Hypomyces subiculosus. It contains a cis-enone group that reacts with a cysteine amino acid located right before the conserved DFG motif of the kinase activation loop. Hypothemycin was observed to chemically modify 18 out of the 19 kinases it was tested against, forming a covalent bond. An analysis of the human kinome using bioinformatics identified 46 kinases that possess a specific cysteine residue. Additionally, there are around 200 different kinases that have a cysteine residue near the ATP pocket. This suggests that a significant number of extra kinases could potentially be targeted by irreversible inhibitors. Nevertheless, despite the considerable quantity of kinases that may be potentially suppressed by this method, some drug developers express apprehension over the potential toxicity of covalent inhibitors due to the alteration of unforeseen targets.

#### Monoclonal antibodies

Monoclonal antibodies (mAbs) have emerged as a powerful and versatile class of targeted therapies in the field of cancer treatment. These antibodies are designed to specifically recognize and bind to certain proteins or receptors that are over-expressed on the surface of cancer cells, thereby exerting their therapeutic effects through various mechanisms, including immune-mediated cytotoxicity, blockade of signaling pathways, and targeted delivery of cytotoxic agents to the tumor cells [120, 146]. Monoclonal antibodies in cancer therapy work by triggering antibody-dependent cellular cytotoxicity (ADCC) along with complement-dependent cytotoxicity (CDC) [121]. Monoclonal antibodies can attach to certain antigens on cancer cells, which then attract immune cells like natural killer (NK) cells as well as macrophages to the tumor area. This leads to the elimination of cancer cells by releasing toxic substances and activating the complement system [147]. Additionally, monoclonal antibodies can function as antagonists by blocking critical signaling pathways that promote cancer cell growth and survival. For example, trastuzumab, a monoclonal antibody targeting the human epidermal growth factor receptor 2 (HER2), inhibits the HER2 signaling pathway, thereby suppressing the proliferation and metastasis of HER2-positive breast cancer cells [148]. Furthermore, it is possible to manipulate monoclonal antibodies to transport deadly substances directly to cancerous cells, resulting in their targeted eradication while minimizing harm to normal cells. Antibody–drug conjugates (ADCs) represent a notable advancement in this area, where monoclonal antibodies are conjugated to potent cytotoxic payloads, such as chemotherapy drugs or toxins. Upon binding to the target antigen, the ADC is internalized by the cancer cells, leading to the release of the cytotoxic payload and subsequent cell death [149]. Despite their therapeutic potential, the clinical utility of monoclonal antibodies can be influenced by the development of resistance mechanisms, as well as the potential for adverse effects and immunogenicity. The emergence of resistant cancer cell populations can result from the downregulation or mutation of the target antigen, leading to the attenuation of the antibody's efficacy over time [150]. Furthermore, the activation of alternative signaling pathways can enable cancer cells to bypass the inhibitory effects of monoclonal antibodies, contributing to disease progression and treatment failure. To address these challenges, ongoing research efforts are focused on the development of combination strategies that combine monoclonal antibodies with other targeted therapies or immune checkpoint inhibitors to enhance their efficacy and overcome resistance mechanisms [151]. Additionally, the refinement of antibody engineering techniques and the identification of novel target antigens aim to expand the applications of monoclonal antibodies to a broader range of cancer types, further improving their therapeutic potential and clinical outcomes for cancer patients [152].

##### Antibody structure and function

A member of the immunoglobulin (Ig) class, antibodies are bulky glycoproteins that help the immune system identify and destroy foreign antigens. Two heavy chains and two light chains form a fundamental Y shape in their construction [120].

The antigen-binding (Fab) fragments, which are located at the tips of the Y-shaped regions of the antibody, are what recognize the antigen. Immune system components interact with antibodies through the fragment crystallizable (Fc) region at the Y structure’s base. Many different types of immune cells have Fc receptors (FcRs) that allow them to identify antibody Fc regions. Five different types of antibodies may be categorized according to the kind of heavy chain: IgE, IgA, IgG, IgD, and IgM [153]. IgG is the most common type of Ig used in antibody therapy because it mediates specialized functions like antibody-dependent cellular cytotoxicity (ADCC) and complement-dependent cytotoxicity (CDC) by interacting with the associated type of FcR, FcγR, which is present on NK cells, neutrophils, monocytes, dendritic cells, and eosinophils. The capacity of the Fc region to mediate these activities allows for further subclassification of the IgG class: although IgG2 and IgG4 are unable to induce ADCC and CDC, IgG1 and IgG3 can. A monoclonal antibody is a clonal variant of an antibody isotype that has been engineered to specifically target a particular antigen epitope [153].

### Precision medicine and genomics profiling

Precision medicine is a revolutionary healthcare method that seeks to customize medical procedures and therapies based on the distinct characteristics of each patient. In the context of cancer, precision medicine utilizes genomic profiling, which involves the comprehensive analysis of a patient’s genetic makeup, including DNA sequencing, gene expression patterns, and mutational analysis, to identify specific genetic alterations that drive tumor growth and progression [154]. By understanding the molecular and genetic alterations underlying a patient's cancer, clinicians can make more informed decisions about the most effective and targeted treatment strategies, ultimately improving patient outcomes and minimizing the risk of adverse effects. Genomic profiling plays a pivotal role in guiding the selection of appropriate targeted therapies and personalized treatment regimens based on the specific genetic alterations present in the patient's tumor [155]. For instance, the identification of specific mutations or genetic markers, such as epidermal growth factor receptor (EGFR) mutations in non-small cell lung cancer (NSCLC) or BRAF mutations in melanoma, can inform the choice of targeted therapies that specifically inhibit these aberrant pathways, leading to more effective and tailored treatment approaches [156]. Next-generation sequencing (NGS) technology has greatly enhanced genomic profiling by enabling the efficient and affordable sequencing of extensive portions of the genome. This has facilitated the discovery of new genetic changes and possible targets for therapy. NGS can reveal in-depth molecular profiles of tumors, providing valuable information about the genetic diversity and intricacy of cancer. This data can be used to guide the creation of tailored and accurate treatment approaches [157]. Liquid biopsies, another essential component of precision medicine, involve the analysis of circulating tumor DNA (ctDNA) or circulating tumor cells (CTCs) in the bloodstream, providing a non-invasive and real-time assessment of tumor dynamics and genetic evolution. Liquid biopsies offer a minimally invasive alternative to traditional tissue biopsies and can be used to monitor treatment response, detect early signs of treatment resistance, and guide the selection of appropriate targeted therapies, particularly in cases where obtaining tissue samples is challenging or not feasible [158]. Despite its tremendous potential, the implementation of precision medicine in cancer treatment is not without challenges. One of the significant challenges is the interpretation of the vast amounts of genomic data generated from genomic profiling, as the identification of clinically relevant genetic alterations and the determination of their implications for treatment response can be complex and nuanced [159]. Furthermore, the need to address concerns regarding data privacy, regulatory frameworks, and the incorporation of genetic data into regular clinical practice requires continuous endeavors to establish standardized guidelines and protocols for the appropriate and ethical use of genomic data in patient care [160].

### Epigenetic therapies

Epigenetic treatments are a new and hopeful strategy for treating cancer. They target the change of the epigenetic processes that control gene expression, without changing the actual DNA sequence. Epigenetic changes, such as DNA methylation, modifications to histones, and remodeling of chromatin, have significant impacts on the growth and advancement of cancer. These changes affect multiple cellular functions, including cell division, specialization, and programmed cell death. Disruption of these epigenetic processes can result in the abnormal activation of genes associated with tumor development and spread, rendering them appealing targets for therapeutic intervention [161]. One of the key strategies in epigenetic therapy involves the use of DNA methyltransferase (DNMT) inhibitors, which work by blocking the activity of DNMT enzymes responsible for adding methyl groups to specific DNA sequences, thereby leading to the reactivation of tumor suppressor genes that were silenced through aberrant DNA methylation. For instance, hypomethylating agents, such as azacitidine and decitabine, have demonstrated efficacy in the treatment of myelodysplastic syndromes (MDS) and acute myeloid leukemia (AML), leading to the expression of silenced tumor suppressor genes and the induction of cell differentiation and apoptosis. Histone deacetylase (HDAC) inhibitors represent another important class of epigenetic therapies, functioning by inhibiting the activity of HDAC enzymes responsible for removing acetyl groups from histone proteins, resulting in chromatin relaxation and the transcriptional activation of genes involved in cell cycle regulation and apoptosis. HDAC inhibitors have shown promise in the treatment of various hematological malignancies and solid tumors, such as cutaneous T-cell lymphoma (CTCL) and multiple myeloma, by promoting tumor cell differentiation, inducing cell cycle arrest, and enhancing the immune recognition of cancer cells [162, 163]. Despite their therapeutic potential, the clinical utility of epigenetic therapies can be influenced by certain challenges, including the development of resistance mechanisms, non-specific effects on gene expression, and the potential for adverse events, such as myelosuppression and gastrointestinal toxicity. The objective is to improve the effectiveness of cancer treatments and overcome resistance mechanisms by identifying predictive biomarkers and developing combination strategies that integrate epigenetic therapies with other targeted agents or immune-mediated checkpoint inhibitors. This will ultimately lead to better clinical outcomes for patients with different types of cancer. Furthermore, ongoing research efforts are focused on elucidating the intricate mechanisms of epigenetic regulation in cancer, identifying novel epigenetic targets, and developing more selective and potent epigenetic agents that can effectively modulate gene expression patterns and restore the normal epigenetic landscape in cancer cells. These efforts have the potential to progress the field of epigenetic therapies and offer new opportunities for the creation of improved and tailored treatment methods for individuals with cancer [164].

#### DNA methylation

The majority of research on epigenetic changes has focused on DNA methylation in mammals. The dinucleotide CPG is methylated in promoter genes [165]. DNA methyltransferases aid in the methyl group transfer from S-adenosyl methionine (SAM) to the C5 site of cytosine in DNA methylation. This results in gene silence, which, since its transcription is hampered, reduces gene expression and activation. Cell division confers these methylation areas heritably. Once methylated DNA is bound by the repressor Mecp2, a complex including histone deacetylase and a transcription repressor is recruited. Gene sequence affinity for transcriptional factors then decreases. Tumor suppressive gene transcription is therefore inhibited. This gene controls cell division, inhibits cancer, and causes DNA to repair itself [166]. Additionally able to attach to cytosine in methylated form, methyl CPG binding protein inhibits the binding of other transcription-related proteins. It binds to histone deacetylases, which attach to methylated sites. After that, some histones and proteins are not acetylated [167]. The methylation regions of DNA are not bound by transcriptional factors when methylated. Lowering of binding affinity is another possibility. Methylation gone wrong can lead to cancer. Regulating 600 genes is the responsibility of epigenetic processes. Tumors may not just result from methylation of DNA. The hypermethylated gene and DNA methylation may be the reason for gene silence. Among the several kinds of histone modifications, methylation of cytosine is the most exclusive modification of DNA [168].

#### DNA methyltransferases (DNMTs/DNA MTase)

Methyltransferases of DNA are analogs of enzymes that facilitate the methyl group transfer to DNA. One methyl donor used by all DNA methyltransferases is called S-adenosyl methionine (SAM). Through interactions with histone deacetylases and other proteins with transcriptional repression characteristics, DNMTs directly decrease transcription. DNMTs silence genes either with or without DNA methylation [169]. Three DNA methyltransferases are in an active state in mammals. These are DNMT1; DNMT3A; and DNMT3B. The fourth enzyme DNMT2 is not DNA methyltransferase. Methyltransferases are classified as either maintenance or denovo methylation methyltransferases [170]. De novo methyltransferases conduct the new cytosine methylation. Usually, they exhibit themselves early in embryonic life. When one strand of DNA is methylated previously, maintenance methyltransferases methylate the other. They keep an organism in the methylation pattern that de-novo methylation established all of its life. The enzyme most commonly found in human cells is called DNMT1. DNMT1 maintains the pre-existing methylation in both normal and cancerous cells. Reacting on hemimethylated DNA, DNMT1 duplicates the methylated pattern in the freshly produced DNA strand [171]. Hemi-methylated DNA has a 7–100 times higher in vitro activity of this enzyme than unmethylated DNA. Human cancer cells use DNMT1 to maintain and de-novo methylate tumor suppressor genes. 1620 amino acids make up DNMT1 [172]. It includes catalytic and regulatory domains. Catalytic activities need both domains. DNMT3 enzyme does de novo methylation. Hemi-methylated and finally fully methylated CPG are formed by the methyl group being added to the CPG sequence by DNMT3. It so proceeds to methylate hemi- and unmethylated CPG at a similar rate. DNMT3A, DNMT3B, and DNMT3L are its three members. While it happens more quickly than DNMT1 but much more slowly than DNMT3A, CPG dinucleotide methylation happens via the enzyme DNMT3A. In structure, DNMT3L is very similar to DNMT3A and DNMT3B. Although inactive by itself, it is essential for DNA methylation. The sequences of DNMT2 (TRDMT1) and 5-methylcytosine methyltransferases are identical. Though it methylates aspartic acid tRNA at position 38, this enzyme is not in charge of methylating DNA. To better reflect its many roles, it was renamed TRDMT1 (tRNA aspartic acid methyltransferase 1) [173].

#### Histone modifications

Alteration of histones takes place in the N-terminal region rather than in the body of the histone because the N-tail region is more accessible. The methylation of histone H3 at lysine 4 (H3K4) occurs in active genes, whereas it occurs at lysine 9 and 27 in inactive genes [174, 175]. Principal transcriptional modifications include phosphorylation, methylation of H3K9, and hypoacetylation of H3. These are the ones who silence genes and alter the structure of chromatin. Marking active transcribing areas is done by histone acetylation. Areas with deacetylation of histones are transcriptionally silent. Marking of both active and inactive regions is done via histone methylation. However, histone modification can also initiate DNA methylation, and DNA methylation can initiate histone modification. While histone deacetylase eliminates the acetyl group and is in charge of histone deacetylation, histone acetyltransferases (HATs) are in charge of hypoacetylation of histone at lysine residues [176]. Condensing of chromatin results from this, which also silences genes and prevents transcription. Histone deacetylation causes chromatin condensation. Melt CPG binding protein is the cause of histone deacetylase recruitment. Some additional proteins and histones are deacetylated by HDACs. Cancer is developed by the silencing of the gene. Furthermore, activating DNMTs are HDACs. On histone, lysine 27 and 9 methylation also inhibit transcription and silences genes. Furthermore, recruited by DNMTs are histone deacetylases. Increased DNA methylation may potentially be the result of histone-mediated suppression of cancer-suppressing genes. Generating silence is their joint responsibility. Histone acetylation is linked to gene activation; histone deacetylation to gene inactivation [177].

#### RNA associated silencing/chromatin remodeling

Transcriptional and post-transcriptional gene silencing are two categories of RNA-associated gene silencing [170]. In post-transcriptional gene silencing, RNA creates heterochromatin and results in heritable transcriptional gene silencing [178]. Anti-sense transcripts, non-coding RNAs, and RNAi (RNA interference) are examples of RNA transcriptional gene suppression. Furthermore, increasing DNA methylation and histone alterations is RNA-associated silencing [179]. But antisense RNA has been shown to silence genes. In αthalassemia, antisense transcription is what silences the globin gene and methylates DNA [180].

#### Genomic imprinting

The process is known as specific allele silencing [181]. A single paternal gene is silenced in this case, while the other genes remain unaffected [182]. It stays put because of methylation regions around or inside the imprinted gene [183].

#### Other factors responsible for epigenetic variations

DNA methylation pattern dysregulation can be caused by environmental factors such as hormones and cigarette smoke [175]. Malignancy sets in. Diethyl stilbesterol is one of several xenobiotics that alter methylation patterns and cause gene expression abnormalities [170]. The methylation pattern and tumor development can be influenced by nutritional variables as well [184].

#### DNA methylation role in prognosis and prevention

Alterations to DNA methylation serve several purposes in a therapeutic context. Oncogenic cells exhibit hypermethylation of genes [170]. A CPG island that is not methylated is found in normal cells. It is possible to evaluate cancerous alterations by keeping these in mind [185]. Since hypermethylation of genes occurs before these modifications, these methods can be employed to identify cancerous mutations [186]. Clinicians can gain prognostic information from altered methylation patterns. Hypermethylation of the p16 gene, for instance, can identify lung cancer. Several methods exist for detecting methylation [170].

#### Drugs used for epigenetic cancer therapy

The current medications are less effective due to the issues of drug resistance and tolerance. There have been attempts to address this issue by utilizing polymers or nanotechnology to enhance medication distribution to the target region, synthesizing novel pharmaceuticals by proteomics or lactic acid bacteria marine microorganisms, or both [187]. New anti-inflammatory, hypotensive, hepatoprotective, hypoglycemic, amoebicidal, anti-fertility, cytotoxic, antibiotic, spasmolytic, bronchodilator, antioxidant, anti-diarrheal, and anti-Parkinsonism medications are greatly sought for in today's medical technology [188]. In a similar vein, the beneficial benefits of a plethora of anticancer medications are being considered. Considering that epigenetic changes can be reversed, it is possible to address these changes by therapeutic means [189]. Enzyme inhibitors, such as DNA methyltransferases and histone deacetylases, are available in the form of synthetic medicines. In therapeutic settings, synthetic medicines are employed either as single agents or in combination [190].

Given the connection between histone changes and DNA methylation, combination treatment targets both processes. While higher dosages of DNMT inhibitors can be administered alongside HDAC inhibitors, smaller levels can be used alone. One such method of combination therapy is to apply conventional chemotherapy, immunotherapy, or interferon after first treating cancer cells using epigenetic therapy.

Contrary to DNMTs, HDACs by themselves are not in charge of gene hypermethylation. Clinical testing is primarily done for DNA methyltransferase. Cancers of the blood have been treated with DNMTi and HDACi. They work hardly at all on solid tumors. HDACi by itself are unable to express the hyperacetylated gene, which is why DNMTi are dominant over HDACi. Reversing aberrant epigenetic patterns has been proposed to be possible by nutrition. HDACs, MBD, and DNMTs are decreased by dietary variables and epigenetic treatments; these agents also alter DNA methylation status and restore histone in an acetylated state. Methylene marks at the promoters of dormant genes can also be increased by them. After that, transcriptional factors will have access to the gene to initiate transcription. The most often occurring epigenetic changes are those to DNA and histones. Treatment of breast cancer needs interdisciplinary approaches. Combining surgery, radiation, cytotoxic chemotherapy, and molecular targeted therapy are among the treatment options for breast cancer. Nowadays, epigenetically regulated changes are the main focus of therapy changes. The activity of certain enzymes called DNA methyltransferases and histone deacetylases determines these changes. These days, it is thought that epigenetic therapy should target these enzymes. In epigenetic treatment, inhibitors of these enzymes exhibit anticancer properties in malignant illnesses [173].

##### DNA methyltransferase inhibitors

The gene expression in the gene regulatory regions is influenced by DNA methylation. Five-hydroxymethylcytosine is a novel DNA modification that results in the demethylation of DNA in the genome of mammals [191]. Transcriptional gene inactivation is caused by hypermethylation of the CPG islands. Many times, the promoter regions of genes have hypermethylation. These areas are concerned with apoptosis, DNA repair, and cell cycle control. One of the numerous cancers where CPG island DNA hypermethylation has been found is myelodysplastic syndrome. Leukemia of myelogenous origin [171]. The enzymes known as DNA methyltransferases are responsible for establishing and preserving DNA modification. Mammal active DNA methyltransferases are DNMT1, DNMT3A, and DNMT3B. The methylation pattern of DNA is maintained in mammalian cells by DNMT1 activity. A methylation pattern is denovo established by DNMT3A and DNMT3B. The inability to preserve methylation during DNA replication or the separate processes of base excision repair (BER) and nucleotide excision repair (NER) can both result in the demethylation of DNA [192]. Demethylation results via the iron-dependent α-ketoglutarate dioxygenase enzyme TET1’s conversion of 5-methylcytosine to 5-hydroxy methylcytosine. The primary emphasis of current therapeutic development is on DNMT inhibitors, which include antisense nucleotides, nonnucleoside analogs, and nucleosides. After DNMTs gene inhibition, which has been suppressed during the carcinogenic process by DNA methylation, can be reactivated and the non-carcinogenic condition of the cell restored.

Many tumors are treated using DNMTs inhibitors, which are not particular to any one kind of cancer. Three categories make up DNMTs according to their structure [193].

Inhibition of DNA synthesis is the effect of nucleoside analogs. After transforming a nucleotide, they are incorporated into DNA. They deplete DNMTs by forming covalent compounds with them, and methylation patterns subsequently turn the tables on each other. Five different kinds of nucleoside analogs have been well described. These include 5-azacytidine, 5-aza-2-deoxycytidine (5-azacdr), 5-fluro-2-deoxycytidine, zebularine, and decitabine.

***5-azacytidine*** A prototype DNMTi, 5-azacytidine, has the potential to treat myelodysplastic syndrome [194]. Its two anticancer mechanisms are cytotoxicity and DNA methylation. It was included in DNA as well as RNA [195]. 5-aza-CR treatment of mammalian cells results in tRNA and rRNA defects as well as protein synthesis suppression [196]. Cytotoxicity and chromosomal rearrangement result from this [197]. During DNA replication, its cytosine analog is integrated. Dnmt3 B and DNMT1 are both blocked by it [198]. The acetylation of histones H3 and H4 in promoter areas is caused by it. The gene that suppresses cancer is activated. It makes breast tumor cells more responsive to anticancer drugs [199, 200]. This is an inhibitor of DNA methyltransferase. It blocks the function of methyltransferase [173]. Toxic metabolites of the drug are formed [201, 202]. Repressing DNA methylation restores the expression of a dormant gene. It enters DNA after being phosphorylated to a diphosphate level [203]. It alters the histone acetylation status and inhibits post-transcriptional DNMTs methyl CPG binding proteins. When compared to other DNMTi, zebularine has a lower toxicity against breast carcinoma cell lines [204].

***Decitabine*** A prototype DNA methyltransferase inhibitor is also present [205].

##### Non-Nucleoside analogues

A small number of non-nucleoside analogs have been found to prevent DNA methylation [206]. Incorporating them into DNA is not possible [207]. Their binding site is right on the catalytic area of the DNMTs [208].

***RG 108*** It blocks DNMTs in in vitro studies with human cell lines [209]. It triggers demethylation and reactivates cancer suppressor genes [210]. ***EGCG*** One of the polyphenol compounds that may be found in green tea is identified as EGCG. The transcription of tumor suppressor genes is enhanced and DNA methylation is decreased at micromolar concentrations of EGCG [209].

***Psammaplins*** The HDACs and DNMTs are both inhibited by them [211].

***MG98*** The translation of DNMT1 mRNA is inhibited by MGAS. This results in the hybridization of DNMT1 mRNA. MG 98 is not very harmful [212].

***Hydralazine*** The tumor suppressor gene is transcriptionally reactivated by hydralazine. Hydralazine does not exhibit the typical adverse effects of chemotherapeutic drugs and is well-tolerated, according to phase 1 clinical studies [213].

##### Antisense oligonucleotides

These are complementary short sequences of nucleotides that work with Mrna. By blocking mRNA translation, they hybridize and render the molecule inactive [214].

##### Histone deacetylation inhibitors

Tumors undergo differentiation, apoptosis, and growth arrest when HDAC inhibitors are present. To prevent histones from being converted to their acetylated state, histone deacetylation inhibitors work by blocking the enzyme histone deacetylase. This allows for the return of normal functioning to cancer cells by reversing a number of cellular changes [215, 216]. Histones that have been acetylated trigger the activation of genes that would otherwise remain dormant. To prevent cancer, HDAC inhibitors induce hyperacetylation of histones to accumulate. Demethylation of histones is initiated by the enzyme LSD1. The year 2004 saw its discovery. There are four classes of compounds that block histone deacetylation: benzamides, hydroxamic acids, cyclic tetrapeptides, and short chain fatty acids. Combining HDAC inhibitors with chemotherapeutic drugs might be a future option for breast cancer treatment [216].

### Metabolic interventions

Metabolic interventions in cancer therapy represent a rapidly evolving area of research that focuses on targeting the metabolic alterations and dependencies of cancer cells to inhibit their growth and proliferation. Cancer is characterized by a significant alteration in metabolic processes, characterized by the altered utilization of energy sources and nutrients to support the increased bio-synthetic demands of rapidly dividing cancer cells. The comprehension of the distinct metabolic requirements of cancer cells has facilitated the advancement of novel therapeutic approaches that target and disrupt specific metabolic pathways and weaknesses. One of the key approaches in metabolic interventions involves targeting the dependence of cancer cells on specific nutrients, such as glucose and glutamine, for their energy needs and biosynthetic requirements. By limiting the availability of these essential nutrients through dietary modifications or pharmacological interventions, researchers aim to impair the growth and survival of cancer cells while minimizing the impact on normal cells. Furthermore, the exploitation of the Warburg effect, a phenomenon characterized by the increased glycolytic metabolism observed in cancer cells, has led to the exploration of glycolysis inhibitors as potential therapeutic agents [217, 218]. These inhibitors aim to disrupt the glycolytic pathway, thereby reducing the production of ATP and essential metabolic intermediates required for cell proliferation and survival. Examples of glycolysis inhibitors under investigation include 2-deoxyglucose and lonidamine, which have demonstrated potential in preclinical studies for their ability to inhibit tumor growth and sensitize cancer cells to other treatment modalities. Additionally, the targeting of specific metabolic enzymes and signaling pathways involved in cancer metabolism has gained considerable attention as a potential strategy for metabolic intervention. For example, the suppression of crucial enzymes that play a role in the breakdown of fats, including fatty acid synthase (FASN) and acetyl-CoA carboxylase (ACC), has demonstrated potential in preclinical models for their capacity to hinder the growth and spread of tumors. Modulating the activity of these enzymes can disrupt the synthesis of lipids essential for membrane bio-genesis and signaling pathways crucial for cancer cell survival and proliferation. Despite the promising preclinical data, the translation of metabolic interventions into clinical practice remains a significant challenge, as Understanding the metabolic adaptations and dependencies that are unique to each form of cancer is crucial for comprehending the intricate relationship between cancer metabolism and the tumor micro-environment. Moreover, the potential for off-target effects and the impact on normal metabolic processes necessitate careful consideration and optimization of treatment strategies to minimize toxicity and maximize therapeutic efficacy. Ongoing research efforts continue to focus on unraveling the intricate metabolic networks and dependencies of cancer cells, identifying novel metabolic targets, and developing innovative therapeutic agents and combination strategies that can effectively exploit the metabolic vulnerabilities of cancer cells while preserving the normal physiological functions of healthy tissues. These initiatives possess the capacity to fundamentally transform the field of cancer therapy and open up fresh pathways for the advancement of more precise and individualized therapeutic strategies for individuals afflicted with diverse forms of cancer [219, 220].

### Radio-pharmaceuticals

Radio-pharmaceuticals represent a specialized class of drugs that combine a radioactive isotope with a pharmaceutical compound, enabling the targeted delivery of radiation to specific tissues or organs for diagnostic or therapeutic purposes. Radio-pharmaceuticals are of utmost importance in the realm of cancer therapy as they facilitate the localized and accurate administration of therapeutic radiation to cancerous cells, while concurrently reducing injury to adjacent healthy tissues. By employing this targeted approach, treatment regimens may be developed that are both more efficacious and less hazardous in comparison to conventional external beam radiation therapy [221]. In the context of cancer treatment, targeted radionuclide therapy stands as a prominent implementation of radio-pharmaceuticals. This approach entails the combination of radioactive isotopes with targeting molecules, including peptides or monoclonal antibodies, which exhibit selectivity towards cancer cell receptors or tumor-associated antigens. After the radio-pharmaceutical binds to the target, the cancer cells internalize it. This internalization facilitates the direct destruction of cellular components and DNA by the emitted radiation, ultimately resulting in apoptosis and cell death. Several radionuclides, such as iodine-131, lutetium-177, and yttrium-90, have demonstrated efficacy in targeted radionuclide therapy for the treatment of various types of cancer, including thyroid cancer, neuroendocrine tumors, and certain types of lymphoma. These radionuclides emit different types of radiation, such as beta particles, alpha particles, or gamma rays, which have varying penetration depths and tissue-damaging capacities, enabling the selection of appropriate radionuclides based on the specific characteristics of the targeted tumor [222]. In addition to their therapeutic applications, radio-pharmaceuticals are also extensively used in nuclear medicine for diagnostic imaging procedures, such as positron emission tomography (PET) and single-photon emission computed tomography (SPECT). Diagnostic radio-pharmaceuticals, such as fluorodeoxyglucose (FDG) for PET imaging, enable the visualization and characterization of tumor tissues based on their distinct metabolic activities and functional properties. This allows for the accurate staging, monitoring, and assessment of treatment response in cancer patients, facilitating the timely adjustment of treatment plans and the evaluation of disease progression. Despite their significant clinical utility, the use of radio-pharmaceuticals requires careful consideration and adherence to strict radiation safety protocols to minimize radiation exposure to healthcare providers and patients. Furthermore, the optimization of treatment protocols and the development of novel targeting strategies, such as the integration of combination therapies with chemotherapy or immunotherapy, aim to enhance the therapeutic efficacy and expand the applications of radio-pharmaceuticals to a broader range of cancer types [223].

### The synergy of combination therapies

The synergy of combination therapies in cancer treatment represents a comprehensive and multifaceted approach that leverages the complementary mechanisms of different treatment modalities to enhance the overall anti-tumor response, overcome treatment resistance, and improve patient outcomes. By targeting cancer cells through multiple pathways simultaneously, combination therapies aim to maximize therapeutic efficacy while minimizing the risk of acquired resistance and treatment-related toxicities. One of the key strategies in combination therapy involves the integration of traditional treatment modalities, such as chemotherapy and radiation therapy, with novel targeted therapies or immunotherapies. This approach aims to capitalize on the cytotoxic effects of conventional treatments while harnessing the specificity and selectivity of targeted therapies or immunotherapies to achieve a more comprehensive and sustained anti-tumor response [224]. For example, the combination of chemotherapy with immune checkpoint inhibitors has demonstrated improved treatment outcomes in various cancer types, enhancing the immune-mediated destruction of cancer cells and leading to durable responses in a subset of patients. Moreover, the integration of targeted therapies with different mechanisms of action has shown promise in overcoming treatment resistance and improving the efficacy of individual therapies. For instance, the combination of kinase inhibitors with monoclonal antibodies or immune checkpoint inhibitors can effectively disrupt multiple signaling pathways and enhance the immune recognition of cancer cells, leading to synergistic anti-tumor effects and improved patient responses [225]. Similarly, the concurrent use of epigenetic therapies with conventional chemotherapy or targeted agents can modulate the expression of genes involved in tumor growth and sensitize cancer cells to the cytotoxic effects of standard treatments. In the context of immunotherapy, combination strategies that simultaneously target multiple immune checkpoints or activate different components of the immune system have shown considerable potential in augmenting anti-tumor immune responses and improving treatment outcomes. Through the inhibition of inhibitory checkpoints and the stimulation of immune effector cell activation, combination immunotherapies can surmount immune evasion mechanisms utilized by cancer cells. This, in turn, improves the immune system’s ability to identify and eliminate tumor cells. In addition, the incorporation of genomic profiling and precision medicine into combination therapy protocols enables the detection of particular molecular targets and genetic alterations that can direct the development of individualized treatment approaches tailored to the tumor characteristics of each patient. The individualized strategy endeavors to maximize the effectiveness of treatments, reduce the likelihood of unfavorable consequences, and enhance patient reactions to therapy, ultimately resulting in enhanced life outcomes and k-values for individuals diagnosed with cancer as shown in Table 1 [226].

**Table 1:** The synergy of combination therapies in the market

S.No	Drugs/therapy	Cancer therapy	Targets	Pharmacological action	Limitation	Reference
1.	Doxorubicin	Chemotherapy	Breast cancer, Lung cancer, Hodgkin lymphoma, Non-Hodgkin lymphoma, leukemia, Ovarian cancer, sarcomas	Interacts with DNA	Cardiotoxicity	[227]
				Inhibits topoisomerase		
				Disrupts DNA and RNA synthesis	The risk of cumulative toxicity increases with repeated doses, potentially impacting the long-term use of the drug	
				Induces cell death		
2.	Cisplatin		Testicular, bladder, ovarian, and head-neck cancers	Forms DNA cross-links	Nephrotoxicity	[228, 229]
				Inhibits DNA replication and transcription		
					Neurotoxicity	
				Promotes cell death		
				Causes DNA damage		
3.	Paclitaxel and Docetaxel		Breast, ovarian, lung, and prostate cancers	Stabilizes microtubules	Neutropenia (a decrease in white blood cells), increasing the risk of infections	[230, 231]
				Inhibits cell division		
				Induces cell death		
					Hypersensitivity reactions	
4.	Methotrexate		Leukemia, lymphomas, breast, and lung cancers	Inhibits dihydrofolate reductase	Suppress the bone marrow, leading to myelosuppression and an increased risk of infections, anemia, and bleeding	[232]
					High doses of methotrexate can cause renal toxicity	
				Disrupts nucleic acid and protein synthesis		
				Reduces thymidine production		
5.	5-Fluorouracil		Colorectal, breast, stomach, and pancreatic cancers	Inhibits Thymidylate Synthase	Cardiotoxicity	[233]
				Disrupts Thymidine Synthesis	Gastrointestinal toxicity	
				Inhibits DNA Synthesis Causes Cell Death		
6.	X-ray	Radiation Therapy	Bone Cancer, breast cancer, gastrointestinal cancers, head and neck cancer	They interact with tissues and produce images based on differential absorption	X-rays are non-specific and can affect both cancerous and healthy tissues, leading to potential damage to surrounding normal structures	[234]
7.	Gamma ray		Breast cancer, lung cancer, gastrointestinal cancer, head and neck cancer, brain cancer, prostate cancer, Gynecological Cancers, Bone Metastases	Directed toward damaging DNA in targeted cells	Gamma rays penetrate tissues deeply, they may still cause damage to normal tissues along their path	[235]
8.	Proton ray		Pediatric Cancers, Brain and Spinal Cord Tumors, Eye Tumors (Ocular Melanomas), head and neck cancer, thoracic cancer, Prostate Cancer, Bone and Soft Tissue Sarcomas	Protons deposit their energy in a controlled manner within the targeted tissues, damaging the DNA of cancer cells and inhibiting their ability to divide and grow	Limited accessibility	[236]
9.	Electron beams		Skin cancer, breast cancer, Extremity Sarcomas, head and neck cancer, Keloids and Hypertrophic Scars, Superficial Lymphomas, Eye Tumors	Electron beams interact with tissues to deposit energy. Their use in therapy is aimed at damaging cancer cells and inhibiting their growth	Electron beams are effective for treating superficial tumors but may not penetrate deeply enough for certain deeper-seated tumors	[237]
10.	Curative surgery	Surgery	Breast cancer, colorectal cancer, lung cancer, prostate cancer, thyroid cancer, ovarian cancer, testicular cancer, melanoma, bladder cancer, gastro cancer	Remove the entire tumor or cancerous tissue	Curative surgery may not be feasible if the cancer has spread extensively or if the tumor is located in a critical or inaccessible area	[238]
11.	Debulking surgery		Ovarian, pancreatic, and colorectal cancers	Debulking surgery involves the removal of a portion of the tumor when complete removal is not feasible. The goal is to reduce the size of the tumor, alleviate symptoms, and enhance the effectiveness of other treatments	Debulking surgery may not eliminate all cancer cells, and complete removal may not be achievable in some cases	[239]
12.	Palliative surgery		Palliative surgery used for advanced cancers, easing symptoms, improving comfort	Palliative surgery focuses on alleviating symptoms and improving the quality of life for patients with advanced or incurable cancer. It may involve the removal of a tumor or part of it to reduce pain or obstruction	Palliative surgery does not aim to cure cancer but focuses on symptom relief. It may not be suitable for all patients, especially those with a poor overall health status	[240]
13.	Diagnostic surgery		Cancer biopsy and accurate cancer staging	Diagnostic surgery is performed to obtain a sample of tissue (biopsy) for laboratory analysis, confirming the presence of cancer and determining its characteristics	Diagnostic surgery involves some risk, and there is a possibility of complications. It may not be suitable for all patients, particularly those with significant health issues	[241]
14.	Staging and lymph node removal		Staging surgery used for accurate cancer staging, often involves lymph nodes	Staging surgery helps determine the extent of cancer spread, and it often involves the removal and examination of nearby lymph nodes to assess whether the cancer has spread	Staging surgery may not detect microscopic metastases, and the removal of lymph nodes may cause complications such as lymphedema	[242]
15.	Reconstructive surgery		Breast cancer	Reconstructive surgery is performed to restore the appearance or function of an area after cancer removal	Reconstructive surgery may have aesthetic and functional limitations, and it may not be suitable for all patients, especially those with certain health conditions	[243]
16.	Minimally invasive surgery		Gastrointestinal Cancers, Gynecological Cancers, Thoracic cancer, prostate cancer, head and neck cancer, kidney cancer, liver cancer, and pancreatic cancer	Minimally invasive surgery uses small incisions and specialized instruments to perform procedures with less impact on surrounding tissues	Minimally invasive surgery may not be suitable for complex or extensive procedures, and it may have a steeper learning curve for surgeons	[244]

## Most popular resistance mechanisms to various chemotherapeutic agents

Chemotherapeutic agent resistance mechanisms are intricate, including several biological processes, internal and extrinsic variables, and their interactions. For the development of effective strategies to combat drug resistance and enhance treatment outcomes for cancer patients, it is vital to comprehend these mechanisms [245]. Some of the most common forms of resistance are listed below:

### Tumor heterogeneity

Genetic mutations, epigenetic changes, and clonal development within the tumor micro-environment are the main causes of the complicated phenomena of tumor heterogeneity. Fundamentally, the different cellular composition and functional states seen within a tumor mass are reflected in tumor heterogeneity. Because of continuous gnomic instability, sub-clones with different genetic profiles and behavioral traits form, giving birth to this variety [41]. Crucially, tumor heterogeneity includes cellular interactions in the micro-environment, transcription patterns, and epigenetic control in addition to genetic diversity. Uncovering the complexity of drug resistance and developing individualized treatment strategies that target certain biological vulnerabilities across different tumor populations depend on an understanding of the dynamic character of tumor heterogeneity [245].

### Tumor micro-environment interactions

Cancer development and response to treatment are both affected by the ever-changing tumor micro-environment. A web of signaling pathways and environmental cues is formed when cancer cells interact with stromal cells, immune cells, and components of the extracellular matrix. Cancer cells can develop adaptive responses that enhance their survival and resistance to therapy when exposed to hypoxia, acidosis, and nutritional restriction in the tumor micro-environment [246]. Furthermore, chemo-resistance can be conferred by paracrine communication between tumor and stromal cells, which activates pro-survival pathways such as PI3K/AKT and MAPK signaling. To discover novel therapeutic targets and solutions to the problem of drug resistance, it is critical to analyze the complex interplay between tumor cells and their microenvironment [247].

### Cancer stem cells (CSCs)

Some tumor cells have stem-like characteristics, such as the ability to self-renew, differentiate, and start tumors. These cells are called cancer stem cells (CSCs). Tumor heterogeneity and therapeutic resistance are profoundly impacted by these cells, which are intrinsically plastic and resistant to traditional treatments. CSCs interact with extracellular matrix components and stromal cells to preserve their stem-like characteristics and advance the tumor micro-environment [248]. These cells inhabit specialized niches. Crucially, CSCs are resistant to radiation and chemotherapy because they have increased efflux transporter activity, DNA repair pathways that are activated and apoptosis resistance. A promising strategy for eliminating treatment-resistant tumor populations and avoiding disease recurrence is to target CSCs [249].

### Inactivation of anticancer drugs

Drug inactivation and detoxification are two of the many ways cancer cells avoid the cytotoxic effects of anticancer medications. Chemotherapeutic drugs are rendered inactive or less lethal by enzymes such as cytochrome P450s and glutathione S-transferases (GSTs), which catalyze their metabolism and conjugation. Additionally, cancer cells can decrease the effectiveness of chemotherapy by down-regulating drug-activating enzymes or upregulating drug-metabolizing enzymes. Drug resistance in cancer is complicated and requires the development of combination treatment techniques to overcome mechanisms. This is due to the interplay between drug metabolism, cellular signaling pathways, and drug transport systems.

### Multi-drug resistance (MDR)

A significant challenge in cancer treatment is multi-drug resistance (MDR), which occurs when cancer cells develop resistance to anticancer medications that differ in structure and function. The over-expression of ATP-binding cassette (ABC) transporters, including P-glycoprotein (P-gp), MRP1, and BCRP, is the driving force behind this phenomenon [41]. These efflux pumps actively expel chemotherapeutic drugs from cancer cells, decreasing intracellular drug concentrations below lethal limits. To further circumvent the cytotoxic effects of chemotherapy, cancer cells may use other routes for DNA repair, apoptotic signaling, and drug targeting. The development of specific treatments that evade or impede these resistance pathways, as well as an exhaustive knowledge of the molecular processes driving drug resistance, is essential for overcoming MDR [246].

### Inhibition of cell death pathway

The development of chemotherapy resistance in cancer cells can occur when apoptotic pathways, which are crucial for programmed cell death, are impaired. Both internal and external apoptotic pathways might become dysregulated, leading to resistance. Through the extrinsic route, cancer cells can suppress apoptotic signaling in reaction to ligands for extracellular death by down-regulating death receptors or inhibiting enzymes that signal downstream, including caspases. By using the intrinsic route, cancer cells have the ability to block the outer membrane permeabilization of mitochondria and the release of cytochrome c by up-regulation anti-apoptotic proteins such as Bcl-2 and Bcl-xL and down-regulation pro-apoptotic proteins like Bax and Bak. Further, cancer cells might avoid cell death even when exposed to genotoxic stress if mutations in tumor suppressor genes like p53 prevent DNA damage-induced apoptosis [245]. Methods that reestablish apoptotic signaling or target alternate cell death pathways, including ferroptosis or necroptosis, are necessary to overcome resistance to apoptosis.

### Changing drug metabolism

Metabolic enzymes bio-transform chemotherapeutic drugs, altering their pharmacokinetics and effectiveness. Changes in the metabolism of drugs can affect their clearance, activation, or inactivation, all of which contribute to the development of drug resistance. Enzymes like cytochrome P450s catalyze oxidation, reduction, and hydrolysis processes in phase I metabolism, whereas transferases and hydrolases conduct conjugation reactions in phase II. Enzymes involved in drug activation may be downregulated by cancer cells, lowering the efficacy of drugs, whereas enzymes involved in drug detoxification may be upregulated by cancer cells, improving the efficacy of drugs. Individual variability in medication response and interpatient variations in chemotherapy results can be influenced by genetic variants in drug-metabolizing enzymes. In order to optimize chemotherapy regimens and overcome drug resistance in cancer treatment, it is vital to understand the complicated interplay between drug metabolism, drug transport, and drug-target interactions [41].

### Changing drug targets

The cytotoxic effects of chemotherapy are achieved through the targeting of particular molecular pathways that are vital for the survival and proliferation of cancer cells. Drug resistance can develop when drug targets undergo mutations or changes that decrease drug binding affinity or change downstream signaling. In chronic myeloid leukemia, for instance, tyrosine kinase inhibitors like imatinib might become resistant due to mutations in the tyrosine kinase domain of BCR-ABL. Another way that changes to topoisomerases, such as doxorubicin and etoposide, might affect their effectiveness is by changing their binding sites or expression levels. To counteract medication resistance, researchers need to create next-generation inhibitors that specifically target changed or mutant drug targets and devise combination tactics to target several signaling pathways [250].

### Enhancing DNA repair

Cancer cells undergo cell cycle arrest and apoptosis due to the DNA damage induced by chemotherapeutic agents as a mechanism of cytotoxicity. The genotoxic effects of anticancer medications can be reduced by increased DNA repair pathways, which can lead to chemotherapy resistance. For instance, the DNA lesions caused by platinum-based medications, such as cisplatin, can be removed by the nucleotide excision repair (NER) and homologous recombination repair (HRR) pathways, which in turn reduce the effectiveness of the drug [251]. To further avoid cell death caused by DNA damage, cancer cells may further increase the production of DNA repair enzymes such as alkyl transferases. Strategies that target DNA repair pathways or sensitize cancer cells to genotoxic stress are necessary for overcoming resistance to DNA-damaging drugs. For example, combination treatments that block DNA repair enzymes or impair DNA damage response signaling can be used [252].

### Gene amplification

Cancer cells can become resistant to drugs by a process known as gene amplification, which involves increasing the number of copies of particular genes that are involved in drug metabolism, drug transport, or drug targets. One folate antagonist frequently used in cancer treatment, methotrexate, can be rendered ineffective in cases where the dihydrofolate reductase (DHFR) gene has been amplified [253]. A cancer cell's capacity to withstand chemotherapy and drug-induced cytotoxicity depends on its ability to increase certain genes. When medication resistance is detected, molecular methods like quantitative polymerase chain reaction (qPCR) or fluorescence in situ hybridization (FISH) might help doctors decide which treatments to use and which drugs to combine [254].

### Epigenetic alterations

Epigenetic alterations, including DNA methylation and histone modifications, play a critical role in regulating gene expression and chromatin structure in cancer cells. Aberrant epigenetic changes can lead to silencing of tumor suppressor genes or activation of oncogenes, contributing to cancer progression and drug resistance. For example, hypermethylation of the MGMT promoter can confer resistance to alkylating agents like temozolomide by inhibiting DNA repair mechanisms. Similarly, histone deacetylase (HDAC) inhibitors can reverse aberrant histone modifications and restore sensitivity to chemotherapy in drug-resistant cancer cells. Targeting epigenetic regulators represents a promising strategy for overcoming drug resistance and enhancing the efficacy of chemotherapy in cancer treatment [255].

### Additional point

#### Drug efflux pump overexpression

Multidrug resistance is caused by more than just P-gp, MRP, and BCRP; additional efflux pumps including ABCG2 and ABCC1 play a role as well. Cellular homeostasis is disrupted when these pumps are overexpressed, which has two effects: lowering the intracellular concentration of chemotherapeutic drugs and increasing the outflow of endogenous molecules. Mutations in genes, changes in epigenetics, and signals from the microenvironment are all potential drivers of efflux pump overexpression. In addition, CSCs, a kind of tumor cell that can self-renew and differentiate, generally show increased production of efflux pumps, which makes them more resistant to chemotherapy. Strategies to address the underlying processes driving pump overexpression and the development of more effective and targeted inhibitors are necessary to overcome efflux pump-mediated resistance [256].

#### Altered drug metabolism

Chemotherapy resistance is mediated in large part by several drug-metabolizing enzymes and transporters, including cytochrome P450s and glutathione-S-transferases. Drugs can be more easily excreted from the cell when they are conjugated with glucuronic acid or sulfate by phase II conjugation enzymes including UDP-glucuronosyltransferases (UGTs) and sulfotransferases (SULTs). The intracellular accumulation of chemotherapeutic drugs can be influenced by changes in drug uptake transporters such OCTs and OCTNs. In addition, the tumor microenvironment, especially low oxygen levels and acidic pH, might influence how drugs are metabolized and transported, which in turn increases resistance. To combat changed drug metabolism, one must first have an in-depth knowledge of the metabolic pathways at work and then devise tactics to attack several nodes at once [246].

#### DNA repair mechanisms activation

Chromosome resistance is influenced by additional DNA repair pathways besides base excision repair, nucleotide excision repair, and homologous recombination. These pathways include non-homologous end joining (NHEJ) and mismatch repair (MMR). Mutations in essential genes involved in DNA repair, changes to the signaling pathways that respond to DNA damage, or variations in the amounts of repair protein production can all lead to dysregulation of these mechanisms [252]. Apoptosis, cell cycle control, and DNA repair all interact with one another, which can affect how cells react to chemotherapy. One potential strategy for circumventing resistance caused by DNA repair is to take advantage of synthetic lethality, in which the targeting of two supplementary DNA repair pathways results in cell death. To further improve treatment efficacy, researchers are now exploring combination treatments that include DNA-damaging chemicals and inhibitors of certain repair enzymes [251].

#### Target alterations or mutations

Targeted therapy resistance can result from mutations in the target protein as well as changes in downstream signaling molecules and compensatory mechanisms. To illustrate the point, targeted medicines that aim to block EGFR or HER2 can be sidestepped by activating other receptor tyrosine kinase (RTK) pathways like MET or HER3. In addition to the targeted route, changes in the tumor microenvironment, such as enhanced angiogenesis and immune evasion, can bolster tumor growth and survival [246]. To circumvent mutations or changes in targets, researchers need to learn more about the molecular pathways that cause resistance and come up with combination treatments that hit numerous signaling nodes at once. To further extend treatment results, researchers are investigating methods to either avoid or postpone the development of resistance, such as medication vacations and irregular dosage regimens [41].

#### Apoptosis evasion

Cancer cells can develop both intrinsic and extrinsic resistance mechanisms, which provide them an edge in the fight against death. Inhibiting caspase activation and promoting cell survival can be achieved, for instance, by upregulating anti-apoptotic proteins like XIAP and surviving. In addition, the apoptotic response to DNA damage induced by chemotherapy can be compromised by dysregulation of the tumor suppressor protein p53, which double-checks apoptosis. Additionally, the apoptotic machinery can be compromised by changes in the expression or activity of pro-apoptotic BH3-only proteins, including Bim and Bad. In addition to the identification of novel targets implicated in apoptotic signaling pathways and the development of BH3 mimetics that restore apoptotic sensitivity, a multifaceted strategy is required to overcome apoptosis evasion. Furthermore, approaches aimed at manipulating the tumor microenvironment and augmenting immune-mediated cell death exhibit potential in surmounting cancer cell resistance to apoptosis [257].

## Case studies: successful novel therapeutic agents

### Inspiring breakthroughs and success stories

The landscape of medicine has been profoundly shaped by groundbreaking innovations, and case studies of successful novel therapeutic agents stand as testaments to the trans-formative power of scientific discovery. These inspiring breakthroughs not only exemplify the triumphs of research and development but also underscore the tangible impact on patient care and outcomes [258].

#### Imatinib (Gleevec) in Chronic Myeloid Leukemia (CML)

Imatinib, an inhibitor of tyrosine kinase, brought about a paradigm shift in the management of Chronic Myeloid Leukemia (CML). Before its introduction, CML had limited treatment options and often progressed to an advanced and life-threatening stage. Imatinib selectively targets the abnormal protein produced by the BCR-ABL gene, which is characteristic of CML. The drug’s introduction marked a paradigm shift, transforming CML from a largely untreatable condition to a manageable chronic disease. m not only extended patient survival but also served as a model for precision medicine, demonstrating the potential of targeted therapies tailored to the underlying molecular mechanisms of a disease [259, 260].

#### Pembrolizumab (Keytruda) in Immunotherapy

In the treatment of numerous malignancies, pembrolizumab, a monoclonal antibody that specifically targets programmed cell death protein 1 (PD-1), serves as an illustration of the efficacy of immunotherapy. Pembrolizumab, which has received approval for the treatment of melanoma, lung cancer, and various other malignancies, induces an immune response against cancerous cells. The key to its efficacy is its capacity to obstruct the PD-1 pathway, thereby enabling the immune system to more efficiently identify and eliminate cancer cells. The therapeutic revolution that ensued with the drug’s approval of immunotherapies over conventional chemotherapy ushered in a new era in cancer treatment, characterized by long-lasting effects and enhanced patient quality of life [261].

### Navigating challenges and setbacks

Behind the success stories of novel therapeutic agents lie tales of perseverance, resilience, and the adept navigation of challenges and setbacks. While breakthroughs in medicine are celebrated, it is equally crucial to explore the hurdles overcome during the development and deployment of these trans-formative treatments.

#### Imatinib (Gleevec) in chronic myeloid leukemia (CML)

Imatinib's success in treating CML was not without challenges. Over time, some patients developed resistance to the drug, leading to disease progression. Researchers responded by developing second-generation tyrosine kinase inhibitors, such as dasatinib and nilotinib, offering alternative treatment options. This adaptive approach to overcoming resistance showcases the dynamic nature of cancer research and the need for continual innovation in the face of therapeutic challenges [262].

#### Pembrolizumab (Keytruda) in immunotherapy

While immunotherapies like pembrolizumab have revolutionized cancer treatment, they come with unique challenges, notably immune-related adverse events (irAEs). These events, ranging from mild to severe, result from the immune system’s overactivation. Mitigating potential adverse effects while utilizing the immune response against cancer cells is a delicate equilibrium that must be maintained when addressing irAEs. Ongoing research focuses on refining treatment protocols to minimize these events and improve the overall safety profile of immunotherapies.

## Patient-centric approaches in cancer therapy

### The promise of personalized treatment plans

In the dynamic field of cancer therapy, a revolutionary shift towards patient-centric approaches is underway, ushering in an era where treatment plans are meticulously tailored to the unique characteristics of each individual. At the heart of this trans-formative paradigm is the promise of personalized treatment plans, offering a beacon of hope for heightened efficacy and reduced treatment-related side effects [263].

Precision medicine lies at the core of these personalized treatment plans, where clinicians delve into the molecular and genetic intricacies of a patient’s cancer. Advances in genomic profiling empower healthcare professionals to identify specific biomarkers driving cancer growth. Armed with this knowledge, treatment strategies can be precisely tailored, deploying therapies designed to interrupt specific pathways responsible for cancer progression. The advantages of personalized treatment plans are profound, aiming to strike a delicate balance between treatment efficacy and the minimization of treatment-related toxicity [264]. By pinpointing the unique molecular fingerprint of a patient's cancer, clinicians can select therapies more likely to be effective, sparing individuals from unnecessary and potentially harmful treatments. By customizing this approach, treatment outcomes may be optimized, response rates may be increased, and the quality of life for individuals undergoing cancer therapy may be enhanced. A critical aspect of personalized treatment plans is their ability to adapt dynamically to changing circumstances. Given cancer's propensity to develop resistance to treatment, clinicians can recalibrate the treatment plan based on real-time data. This adaptability is crucial in the ongoing battle against cancer, emphasizing the importance of a patient-centric and flexible approach. However, challenges exist, particularly related to access and implementation [265]. Not all patients may have access to advanced genomic profiling, and the interpretation of genetic data requires specialized expertise. Addressing these challenges involves expanding access to cutting-edge diagnostics and investing in training healthcare professionals to interpret and apply genomic information effectively. In this patient-centric model, individuals become integral members of their healthcare teams, contributing actively to treatment discussions and making informed choices based on their values and preferences [266]. Effective communication between healthcare providers and patients becomes paramount, fostering a collaborative environment where the patient’s voice is heard and respected. In the ever-evolving landscape of cancer therapy, the embrace of patient-centric approaches reflects a paradigm shift that place individual needs, characteristics, and preferences at the forefront of treatment strategies. Central to this transformative paradigm is the concept of personalized treatment plans, heralding a new era where medical interventions are finely tuned to the distinct molecular and genetic profile of each patient's cancer [267].The advent of precision medicine has been instrumental in unlocking the potential of personalized treatment plans. Through advanced genomic profiling, healthcare professionals can unravel the intricate details of a patient’s cancer, identifying specific biomarkers that drive its growth [268]. This in-depth understanding allows for the selection of targeted therapies, honing in on the underlying molecular pathways responsible for cancer progression. The significant advantages of personalized treatment plans extend beyond mere efficacy—they aim to strike a delicate equilibrium between treatment effectiveness and the reduction of treatment-related side effects. By tailoring interventions based on the unique characteristics of a patient's cancer, clinicians seek to optimize treatment outcomes while minimizing the burden on individuals, ultimately enhancing their overall quality of life during and after therapy. One of the distinguishing features of personalized treatment plans is their adaptability [268]. Given the inherent ability of cancer to develop resistance to treatments over time, this flexibility becomes a crucial asset. Clinicians can dynamically adjust treatment plans based on real-time data, incorporating new therapies or modifying existing regimens to address emerging challenges [269]. This dynamic approach underscores the resilience required in the ongoing battle against cancer. However, challenges persist in the widespread implementation of personalized treatment plans, particularly related to access and expertise. Not all patients have equal access to advanced genomic profiling, and the interpretation of complex genetic data demands specialized knowledge. Bridging these gaps involves not only expanding access to cutting-edge diagnostics but also investing in the education and training of healthcare professionals to ensure the effective application of genomic insights [270]. In this patient-centric model, individuals assume an active role in their healthcare journey. Collaborative decision-making becomes a hallmark, with patients contributing to treatment discussions and making informed choices aligned with their values and preferences. Effective communication between healthcare providers and patients fosters a partnership built on mutual respect, where the patient's voice is not only heard but is integral to shaping the course of their care [271].

### Enhancing quality of life through supportive care

In the realm of cancer therapy, a holistic commitment to patient well-being extends beyond the targeted precision of treatments. Patient-centric approaches recognize the significance of enhancing the quality of life through supportive care, weaving a tapestry of comprehensive services that address physical, emotional, and practical needs throughout the cancer journey. At the core of patient-centric cancer care is the recognition that the journey involves not only medical interventions but also a nuanced understanding of the challenges individuals face [272]. Supportive care encompasses a spectrum of services designed to alleviate the physical and emotional burdens associated with cancer treatment, fostering a holistic approach that extends beyond the confines of traditional medical care. Supportive care excels in managing the physical aspects of cancer treatment [273]. From mitigating treatment-related side effects such as nausea, pain, and fatigue to providing palliative care for those with advanced disease, these interventions enhance the overall well-being of patients [274]. Holistic pain management, nutritional support, and rehabilitation services contribute to maintaining or improving patients' physical function and comfort. Cancer diagnosis and treatment can exert a profound toll on mental and emotional well-being. Patient-centric care acknowledges the importance of emotional support services, including counseling, psychotherapy, and support groups [275]. These resources provide a space for individuals to express fears, anxieties, and uncertainties, fostering resilience and equipping patients with coping mechanisms that enhance their mental and psychological health. Beyond the medical aspects, cancer patients often grapple with practical challenges such as financial strain, transportation issues, and navigating the complexities of healthcare systems. Supportive care recognizes these practical hurdles and helps through social work services, financial counseling, and access to community resources. By addressing these challenges, patient-centric care seeks to reduce the burdens that extend beyond the treatment room, enabling individuals to focus on their health and well-being [276].

An integral component of supportive care is patient education. Empowering individuals with information about their diagnosis, treatment options, and potential side effects enhances their ability to make informed decisions about their care. Educational programs also promote health literacy, ensuring that patients understand their treatment plans, follow-up care, and the importance of proactive health management [277]. Effective patient-centric care integrates supportive services seamlessly with primary care, creating a continuum of support that extends beyond the confines of cancer treatment centers. Collaboration between oncologists, primary care physicians, and a multidisciplinary team of healthcare professionals ensures that patients receive comprehensive, coordinated care that addresses their diverse needs. In the evolving landscape of cancer therapy, the integration of supportive care stands as a testament to the commitment to elevating the patient experience [278]. Beyond the precision of treatment plans, patient-centric care recognizes the multifaceted nature of the cancer journey. By enhancing the quality of life through supportive care, healthcare providers contribute not only to the physical well-being of patients but also to their emotional resilience and overall satisfaction with their care journey. This holistic approach to patient-centric care extends beyond the clinical setting, emphasizing the importance of creating a supportive ecosystem that acknowledges and addresses the diverse challenges patients may encounter. It recognizes that the impact of cancer extends to every facet of an individual's life, and thus, comprehensive care should extend its reach to social, emotional, and practical dimensions. Supportive care, with its emphasis on managing physical symptoms and addressing emotional well-being, strives to make the cancer journey more navigable. As patients face the complexities of treatment, the inclusion of palliative care services ensures that comfort and dignity are prioritized, particularly in the advanced stages of the disease [279]. By attending to the holistic needs of individuals, supportive care becomes an integral part of fostering resilience and enabling patients to cope with the challenges they encounter. In the realm of mental and emotional support, patient-centric approaches recognize that emotional well-being is as crucial as physical health. Counseling and support groups provide invaluable spaces for patients to share their experiences, fears, and triumphs. This interconnectedness creates a sense of community among individuals who may be going through similar challenges, fostering a support network that extends beyond the healthcare system. Practical assistance, addressing the tangible challenges of financial strain and logistical hurdles, becomes a lifeline for patients navigating the complexities of cancer care. Social work services and financial counseling not only alleviate immediate stressors but also contribute to a more equitable and accessible healthcare experience. By actively addressing these practical challenges, patient-centric care strives to reduce disparities in care and ensure that all individuals, regardless of their circumstances, receive comprehensive support. Education, another cornerstone of supportive care, empowers patients to actively participate in their treatment decisions. Informed patients are better equipped to collaborate with their healthcare teams, ask questions, and actively engage in shared decision-making. Beyond treatment specifics, educational initiatives contribute to broader health literacy, enabling patients to take charge of their overall well-being. The seamless integration of supportive care with primary care services ensures a continuous, patient-centered approach that transcends the confines of specialized treatment centers. This collaboration emphasizes the importance of a united healthcare front, where various professionals work together to provide comprehensive and cohesive care that addresses the unique needs of each patient.

## The future of cancer therapy

A combination of breakthrough technology, early detection tactics, and worldwide collaboration is driving a paradigm change in cancer therapy. Artificial intelligence (AI) and big data are emerging as disruptive forces. AI, aided by machine learning, analyses large datasets to detect detailed patterns, allowing for personalized cancer diagnosis and treatment planning based on individual genetic profiles. This not only improves treatment effectiveness but also represents a big step forward in precision medicine. Concurrently, significant advances in early diagnosis and preventive measures light the cancer therapeutic horizon. Screening technologies and molecular diagnostics have advanced to unprecedented levels, allowing malignancies to be identified in their early stages. Liquid biopsies, which use circulating tumor DNA to identify cancer and track therapy responses, are a non-invasive method of diagnosing cancer and monitoring treatment responses. This trend towards earlier identification not only improves treatment results but also allows for less intrusive procedures, changing the cancer care trajectory.

Global collaboration is essential in this transforming context. Researchers, healthcare experts, and pharmaceutical companies are banding together to speed up the discovery and distribution of innovative cures. Open-access research platforms and worldwide clinical trial networks facilitate the sharing of data and knowledge, allowing scientists to translate scientific advances into clinically successful medicines. Furthermore, worldwide efforts are being made to address healthcare inequities and enhance infrastructure in neglected areas, ensuring that the advantages of cutting-edge cancer medicines reach a wide range of people [280, 281].

## Conclusion

The landscape of cancer therapy is undergoing an unprecedented transformation, driven by a multifaceted array of innovative approaches that promise to reshape the way we understand and combat this complex disease. From the groundbreaking strides in immunotherapy harnessing the power of the immune system through checkpoint inhibitors, CAR-T cell therapy, and cancer vaccines, to the targeted precision of therapies like kinase inhibitors and monoclonal antibodies, the options available for patients and clinicians are expanding at a rapid pace. The integration of precision medicine, fueled by advancements in genomics and molecular profiling, has ushered in an era where treatment strategies can be tailored to the unique genetic makeup of individual patients. Epigenetic therapies, metabolic interventions, radiopharmaceuticals, and the promising synergies of combination therapies further augment this evolving landscape, offering new avenues for therapeutic exploration. Critical to this progression is the rigorous stages of multiple-phase clinical trials, ensuring the safety and efficacy of emerging treatments while providing patients access to novel therapies. These trials, coupled with ethical considerations, regulatory approvals, and vigilant safety monitoring, lay the groundwork for the successful integration of innovative treatments into mainstream cancer care. The success stories arising from novel therapeutic agents underscore the remarkable breakthroughs achieved, but they also underscore the challenges and setbacks inherent in this journey. However, these setbacks serve as opportunities for learning and growth, guiding researchers and healthcare providers toward more refined approaches in cancer therapy. Central to these advancements are patient-centric strategies, emphasizing personalized treatment plans tailored to individual needs and the optimization of supportive care to enhance the quality of life for patients undergoing treatment. The active involvement of patients in clinical trials not only facilitates access to novel therapies but also fosters a sense of empowerment and advocacy within the cancer community. In the future, early detection and preventive tactics might be completely transformed by combining artificial intelligence with big data. Collaborative efforts on a global scale are imperative to ensure improved access to these innovative therapies, transcending geographical boundaries and socio-economic barriers.

## Data Availability

No datasets were generated or analysed during the current study.
